# Revision Rates After Primary ACL Reconstruction Performed Between 1969 and 2018: A Systematic Review and Metaregression Analysis

**DOI:** 10.1177/23259671221110191

**Published:** 2022-08-05

**Authors:** Rasmus J. Liukkonen, Ville T. Ponkilainen, Aleksi Reito

**Affiliations:** † Faculty of Medicine and Health Technology, Tampere University, Tampere, Finland.; ‡ Department of Surgery, Central Finland Hospital Nova, Jyväskylä, Finland.; *Investigation performed at Faculty of Medicine and Health Technology, Tampere University, Tampere, Finland*

**Keywords:** ACL, anterior cruciate ligament, revision, metaregression, traumatology

## Abstract

**Background:**

Numerous studies, including randomized controlled trials (RCTs), have been published on the optimal graft choice for primary anterior cruciate ligament (ACL) reconstruction.

**Purpose:**

To review existing studies to investigate whether advances in orthopaedics have affected revision rates after primary ACL reconstruction.

**Study Design:**

Systematic review; Level of evidence, 4.

**Methods:**

The PubMed database was searched from inception to December 31, 2020, using the PRISMA (Preferred Reporting Items for Systematic Reviews and Meta-Analyses) guidelines. Patient series, observational studies, clinical trials, and registry-based studies investigating primary ACL reconstruction were included, as were high-quality RCTs from an additional study. The minimum required follow-up time for inclusion was 1 year. The primary outcome measure was the pooled prevalence of revision ACL reconstruction. The effect of the year the surgery was performed on revision rates was evaluated with metaregression analysis. All graft types were analyzed simultaneously, and all analyses were repeated separately for each graft type.

**Results:**

Overall, 330 articles with 52,878 patients were included, with a median patient age of 28 years (range, 15-57 years). The primary ACL reconstructions were performed between 1969 and 2018. At a median of 2.3 years of follow-up, the overall revision rate was 3.14% (95% CI, 2.76% to 3.56%); it was 2.71% (95% CI, 2.25% to 3.27%) for hamstring autografts, 2.38% (95% CI, 1.82% to 3.11%) for bone–patellar tendon–bone (BPTB) autografts, and 5.24% (95% CI, 4.02% to 6.80%) for other graft types. For hamstring grafts, the revision rate increased over time (year of surgery), with a 0.0434 (95% CI, 0.0150 to 0.0718) increase effect in the logit-transformed scale for every additional year. There was a slight decrease in revision rates for BPTB (β = –0.0049; 95% CI, –0.0352 to 0.0254) and other graft types (β = –0.0306; 95% CI, –0.0608 to −0.0005) over time; however, confidence intervals for BPTB included the zero change.

**Conclusion:**

Based on this systematic review and meta-analysis, ACL reconstruction is a reliable procedure with overall low historical revision rates. BPTB autograft had the lowest revision rate and a slightly decreasing trend of failures during the past 45 years, although both BPTB and hamstring autografts are reliable graft choices.

Anterior cruciate ligament (ACL) reconstruction is one of the most common orthopaedic procedures.^7^ In recent decades, the incidence of ACL injuries has increased by up to 40%.^15,33^ Increased knowledge of the physiology behind ACL tears and patient-specific differences in prognosis have provided us with a better understanding of options for both treatment and effective postoperative rehabilitation.^16,19,29,31^ Furthermore, shared decision making plays a key role in deciding how to treat an ACL tear to achieve the optimal results.^17,29,31^ ACL reconstruction is the standard for achieving an optimized function, and most patients can return to their preinjury activity level after surgery.^1^

Arthroscopically assisted ACL reconstruction gained popularity in the 1980s, but it was initially considered a challenging and complex procedure.^27^ In the early years, the patellar tendon was the gold standard for reconstruction.^2,27^ Other biological graft origins as well as synthetic materials have been researched for decades, but optimal synthetic materials have not been found, as many of them have been associated with high failure rates.^26^ Surgical techniques have since evolved a great deal because of the increased volume of ACL reconstructions. Today, ACL reconstruction is a common orthopaedic procedure that often leads to good patient-reported outcomes. However, revision surgery after primary ACL reconstruction is needed for various reasons, including reinjury, technical errors in surgery, or instability, with graft failure being the most severe complication.^9,10^

More than 16,000 articles can be found in the PubMed database by searching “ACL reconstruction.” Of these studies, more than half have been published in the past decade. ACL graft type has been a widely researched topic. Research has also focused on different surgical techniques, such as graft fixation methods, different surgical techniques, and optimal timing of surgery. Research has an effect on our treatment methods and options to treat the torn ACL, and currently the treatment for torn ACL may be highly individualized. Research has also affected the worldwide trends in ACL surgery, and currently the torn ACL is usually reconstructed with the use of either hamstring or patellar tendon as a graft.

At the patient level, medical research should have a positive effect on the treatment results. For a single patient, the increasing number of studies during the past few decades should be seen as a better prognosis (ie, faster recovery or decreased failure rate). The revision rate is one of the most important outcomes of ACL reconstruction. To the best of our knowledge, previous studies have not investigated whether revision rates after primary ACL reconstruction have evolved during the recent decades.

The aim of the current study was to investigate whether advances in orthopaedics have affected revision rates after primary ACL reconstruction. Our hypothesis was that the revision rates have decreased within the period studied.

## Methods

### Information Sources and Search Strategy

The PubMed database was searched from inception to December 31, 2020. As a supplementary search, we included randomized controlled trials (RCTs) from the 2017 meta-analysis of Kay et al.^13^ The following search strategy was used: (anterior cruciate ligament or acl or ligamentum cruciatum anterius), (reconstruction* or repair* or surgery* or operation* or reconstructive or graft*), and (fail* or reoperatio* or revision* or re-operation* or retear*). No filters were used. Our review was performed according to the PRISMA (Preferred Reporting Items for Systematic Reviews and Meta-Analyses) 2020 checklist.

### Eligibility Criteria and Selection Process

Records from the database search were imported to a free online systematic review platform (Rayyan; http://www.rayyan.ai), and duplicates were removed. A study was eligible for our analysis if the following inclusion criteria were fulfilled: (1) the focus of the study was primary ACL reconstruction, (2) it was a clinical or registry-based study, and (3) graft ruptures and/or revisions were reported. All the records from the searched database were screened, and abstracts of those articles were assessed by 2 authors (V.T.P. and A.R.). Records meeting the inclusion criteria were selected for the eligibility assessment. After full-text reading, a study was excluded if ≥1 of the following criteria were met: (1) follow-up <12 months; (2) follow-up >10 years; (3) mean patient age <15 years; (4) failures or revisions not reported; (5) non-English language; (6) based on large national registries or similar (eg, Kaiser-Permanente ACL reconstruction registry); or (7) size of the study was <10 patients. Furthermore, if the study included a cohort that had been used in other studies, we included the cohort with the longest or representable follow-up. Studies that were based on national registries or similar were excluded because of the cohorts they might have included that overlapped with those of other, smaller studies with better quality, such as RCTs. The risk of bias assessment was not assessed, because all the studies that met the inclusion criteria and passed the eligibility assessment were included.

### Data Extraction

Data were extracted and recorded in an Excel spreadsheet (Version 16.47; Microsoft). The extracted data included the study characteristics, such as title, authors, publication year, and study setting (ie, RCT, prospective, retrospective). For each study, we collected data on the first year and last year of the included surgeries, duration of follow-up, mean patient age, and number of primary reconstructions. The outcomes included the number of revisions. Other recorded variables were the graft type used in the primary reconstruction and definition of failure. The reason for revision surgery after primary reconstruction was considered the definition of failure. The extracted definitions were graft rupture and graft failure. If only the number of revisions was reported, it was categorized as “revision count.” We only included the number of revisions performed, although not all graft failures were necessarily revised, because the choice to undergo revision surgery is ultimately based on patient preference. However, our aim was to investigate revision rates rather than failure rates.

Graft type used in primary reconstruction was categorized into one of the following categories: hamstring autografts, bone–patellar tendon–bone (BPTB) autografts, or other graft types. Hamstring autografts included all reconstructions that used the semitendinosus and/or gracilis graft. Other graft types included synthetic grafts (eg, Dacron) and autografts other than hamstring or BPTB. If the graft used in primary reconstruction was reported without any specific origin, it was categorized as “other.” Categorization based on surgical techniques (eg, double or single bundle) or autograft and allograft was not conducted, and therefore all the groups might have included both. Study arms were formed based on the subgroups reported in the original article.

### Effect Measures

The primary pooled outcome measure was the prevalence of revision ACL reconstruction. This was calculated by dividing the total number of revisions by the total number of primary reconstructions included in the study. Explanatory variables included in the metaregression were follow-up time, year the surgery was performed, and age at surgery. The year of surgery was calculated as the mean of the first and last years of surgery within each study.

### Data Synthesis and Analysis

All graft types were analyzed simultaneously, and subgroup analyses were conducted separately for each graft type. The proportion of revision ACL reconstruction was determined for each study, and pooled revision rates were reported for each subgroup with 95% CIs. The random-effects model was used because of the high heterogeneity among the analyzed studies. Heterogeneity was assessed using the *I* ^2^ statistic. The fixed-effects model was not used because of inherent patient-specific differences and variability in prognosis. Logit transformation was used to calculate the overall proportions. Metaregression analysis was used to assess whether revision rates decreased as a function of time (year of surgery). A maximum-likelihood estimator was used. If any of the explanatory variables were not reported, the study was not included in the metaregression analysis. The effect of the year of surgery on the revision rate was presented in a scatterplot with a fitted regression line. The weight of the individual studies in the metaregression analysis was presented as the third variable. Finally, we conducted sensitivity analyses based on the duration of the follow-up period, by dividing the follow-up time into different durations and repeating the graft type–stratified metaregression analysis for each of them. All analyses were performed using the meta package from R (Version 4.0.3; R Foundation for Statistical Computing).

## Results

**Figure 1.**
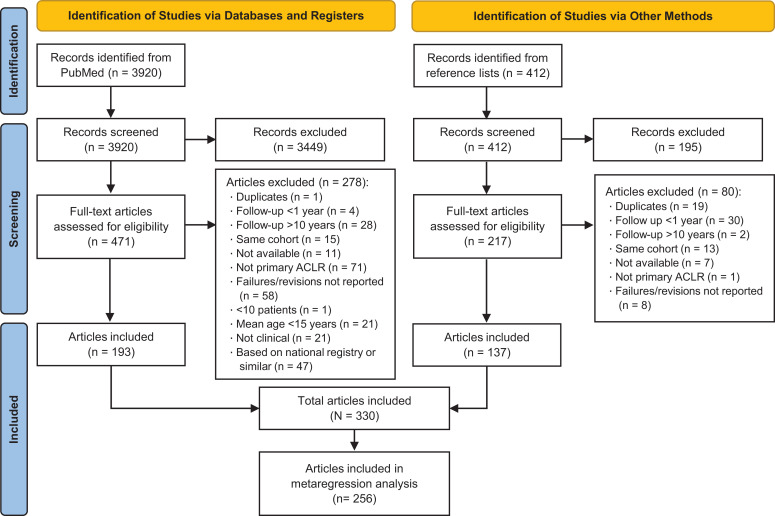
PRISMA (Preferred Reporting Items for Systematic Reviews and Meta-Analyses) flowchart of the study selection. ACLR, anterior cruciate ligament reconstruction.

In total, 3920 articles were found to be eligible for screening. After screening and full-text reading, 193 articles were included. In addition, 137 RCTs from the additional study were included, resulting in a total of 330 articles. The earliest included study was published in 1984 and included primary ACL reconstructions performed as early as 1969,^11^ and the most recent study was published in 2021 and included primary reconstructions performed in 2018.^34^ A flowchart of study inclusion is shown in Figure 1, and publication information for all included articles is available separately as Supplemental Material. A total of 592 study arms with 52,878 patients were included in the study; 101 articles had 1 study arm, 199 had 2 study arms, 27 had 3 study arms, and 3 articles had 4 study arms. The mean age of the participants ranged from 15 to 57 years, with a median age of 28 years. The median number of patients per study arm was 39 (range, 8-6030). The most used graft was hamstring autograft, accounting for 43.2% (n = 22,869) of the patients (Table 1).

**Table 1:** Characteristics of the Analyzed Studies (N = 330) and Pooled Revision Rates*^a^*

Graft Type	Study Arms, n (%)	ACLR, n (%)	Failures, n (%)	Age, y, median (range)	
Hamstring	302 (51)	22,869 (43.2)	1157 (40.1)	28 (15.4-53.9)	
BPTB	148 (25)	9004 (17)	422 (14.6)	28 (15.4-57)	
Other	112 (18.9)	7048 (13.3)	612 (21.2)	29.1 (15-47.1)	
Not reported	30 (5.1)	13,957 (26.4)	694 (24.1)	26.1 (15.1-50.5)	
Total	592	52,878	2885	28 (15-57)	
	Graft Type, n (%)				
Failure Method	Hamstring	BPTB	Other	Not Reported	Total
Graft rupture	662 (57.2)	151 (35.8)	244 (39.9)	449 (64.7)	1502 (52.1)
Graft failure	293 (25.3)	184 (43.6)	273 (44.6)	88 (12.7)	842 (29.2)
Revision count	202 (17.5)	87 (20.6)	95 (15.5)	157 (22.6)	541 (18.8)
Total	1157	422	612	694	2885
Pooled Revision Rate	Failure, % (95% CI)		*I* ^2^, %	Follow-up, y, median (range)	
Hamstring	2.71 (2.25-3.27)		39.7	2.1 (1-10)	
BPTB	2.38 (1.82-3.11)		0.0	2.2 (1-10)	
Other	5.24 (4.02-6.80)		71.1	3 (1-7.8)	
Total	3.14 (2.76-3.56)		59.7	2.3 (1-10)	

*^a^* The reason for revision surgery after primary reconstruction was considered the definition of failure. If only the number of revisions was reported, it was categorized as “revision count.” Definitions of failure were reported as the original author defined them in the analyzed article. ACLR, anterior cruciate ligament reconstruction, BPTB, bone–patellar tendon–bone.

At a median of 2.29 years of follow-up, the overall pooled revision rate was 3.29% (95% CI, 2.91%-3.72%) for all analyzed study arms. The pooled revision rates for the hamstring and BPTB graft types were 2.71% (95% CI, 2.25%-3.27%) and 2.38% (95% CI, 1.82%-3.11%), respectively. For other graft types, the revision rate was 5.24% (95% CI, 4.02%-6.80%). The most common reason for revision surgery was graft rupture (52.1%) (Table 1).

### Metaregression Analysis

**Figure 2.**
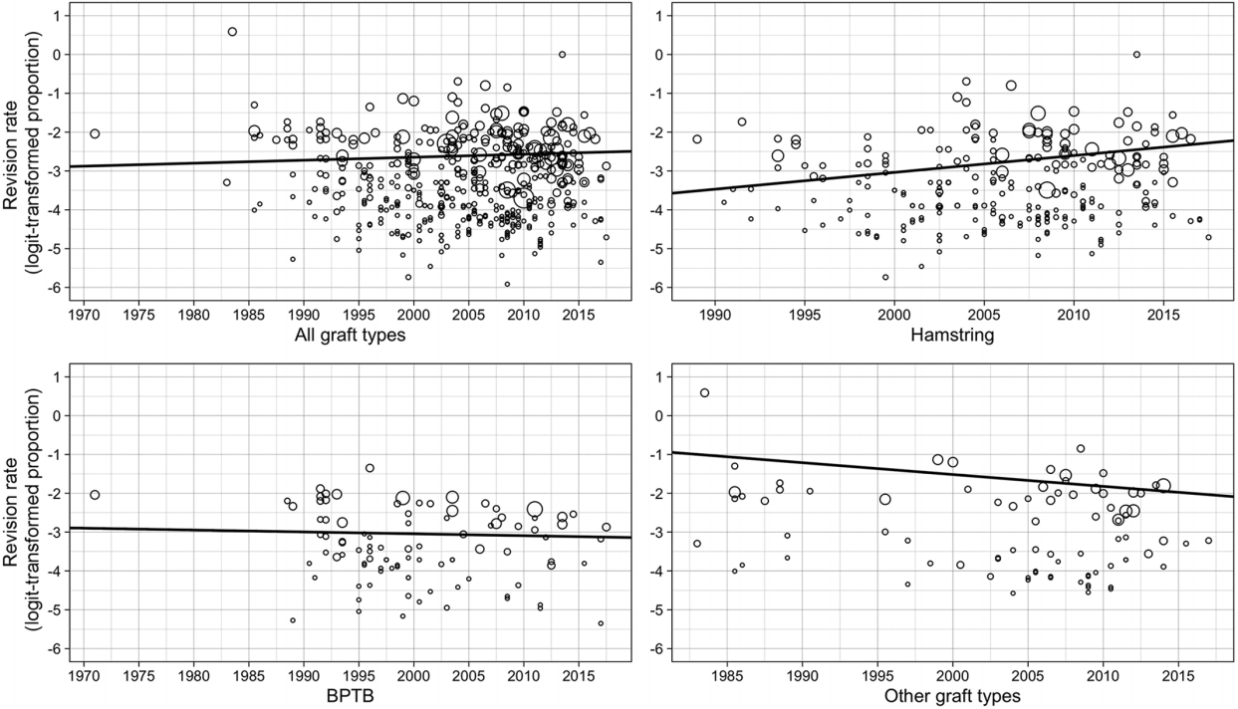
Scatterplot for effect of surgery year with fitted regression line. Single studies and proportions are represented as a function of time on the logit-transformed scale. The weight of the study is presented as the size of the circle. BPTB, bone–patellar tendon–bone.

A total of 460 study arms with 47,653 patients were included in the metaregression analysis: 260 study arms with 20,896 patients for hamstrings, 98 study arms with 7368 patients for BPTB, and 83 study arms with 5541 patients for other graft types. The graft type was unknown in the remaining 13,848 patients and 19 study arms. The year of surgery influenced revision rates, as a 1-year increase in the surgery year had a 0.0077 (95% CI, –0.0084 to 0.0238) increase effect in the logit-transformed scale to the revision rate (Table 2); however, the confidence interval included the zero change. Age at surgery had a decreasing effect with all graft types, as 1 year had a –0.0420 (95% CI, –0.0624 to –0.0216) effect on the logit-transformed scale to the revision rate. The hamstring subgroup was inferior to the BPTB and other graft types, as year of surgery had a clear increase effect (β = 0.0434; 95% CI, 0.0150 to 0.0718) on revision rates, with the confidence interval excluding zero change. The effect for BPTB and other graft types was negative and more imprecise. The effect of year of surgery is shown in the scatterplot in Figure 2.

**Table 2:** Results of Metaregression Analyses*^a^*

	β (95% CI)	SE
All graft types		
Year of surgery	0.0077 (–0.0084 to 0.0238)	0.0082
Mean follow-up	0.1318 (0.0727 to 0.1908)	0.0301
Age at surgery	–0.0420 (–0.0624 to –0.0216)	0.0104
Hamstring graft		
Year of surgery	0.0434 (0.0150 to 0.0718)	0.0145
Mean follow-up	0.1930 (0.1052 to 0.2807)	0.0448
Age at surgery	–0.0499 (–0.0838 to –0.0159)	0.0173
BPTB graft		
Year of surgery	–0.0049 (–0.0352 to 0.0254)	0.0155
Mean follow-up	0.1204 (0.0050 to 0.2358)	0.0589
Age at surgery	–0.0306 (–0.0683 to 0.0070)	0.0192
Other graft types		
Year of surgery	–0.0306 (–0.0608 to –0.0005)	0.0154
Mean follow-up	0.0208 (–0.1598 to 0.2015)	0.0922
Age at surgery	–0.0450 (–0.0876 to –0.0024)	0.0217

*^a^* BPTB, bone–patellar tendon–bone; CI, confidence interval; SE, standard error. .

### Sensitivity Analysis

Sensitivity analyses were performed separately for each graft type. The 2- to 4-year follow-up cohort was the largest in every graft type, including 152 study arms with 11,424 patients with the hamstring graft, 56 study arms with 3686 patients with the BPTB graft, and 58 study arms with 3910 patients with other graft types. BPTB results were more imprecise, and in both 2-year and 2- to 4-year cohorts the effect of the operational year to the revision rates was an increase. The results for other graft types were similar, except for the 2-year cohort, where year of surgery had an increase effect. With the hamstring graft, the results were similar to those observed in the analysis with all follow-up durations. The effect of year of surgery remained the same, with the exception of the 4- to 8-year follow-ups (Table 3).

**Table 3:** Results of the Sensitivity Analyses*^a^*

Follow-up	Hamstring		BPTB		Other Graft Types	
	β (95% CI)	SE	β (95% CI)	SE	β (95% CI)	SE
≤2 y						
Year of surgery	0.0316 (–0.0239 to 0.0871)	0.0283	0.0869 (0.0111 to 0.1626)	0.0387	0.0360 (–0.0537 to 0.1257)	0.7871
Mean follow-up	1.4519 (0.5496 to 2.3542)	0.4604	0.9961 (–0.2331 to 2.2253)	0.6272	–0.1173 (–3.0741 to 2.8395)	–0.0777
Age at surgery	–0.0631 (–0.1259 to –0.0002)	0.0321	–0.0978 (–0.1968 to 0.0013)	0.0505	–0.0972 (–0.2526 to 0.0582)	0.0793
2-3 y						
Year of surgery	0.0630 (0.0183 to 0.1078)	0.0228	–0.0001 (–0.0413 to 0.0411)	0.0210	–0.0300 (–0.0818 to 0.0218)	–1.1359
Mean follow-up	–0.2309 (–1.0395 to 0.5776)	0.4125	0.8264 (–0.0907 to 1.7435)	0.4679	0.7493 (–0.7098 to 2.2083)	0.7444
Age at surgery	–0.0179 (–0.0651 to 0.0294)	0.0241	–0.0493 (–0.1168 to 0.0182)	0.0344	–0.0364 (–0.1117 to 0.0390)	0.0384
2-4 y						
Year of surgery	0.0659 (0.0289 to 0.1029)	0.0189	0.0038 (–0.0315 to 0.0390)	0.0180	–0.0312 (–0.0672 to 0.0049)	0.0184
Mean follow-up	0.1542 (–0.2075 to 0.5159)	0.1845	0.2437 (–0.1887 to 0.6761)	0.2206	0.3661 (–0.1754 to 0.9076)	0.2763
Age at surgery	–0.0304 (–0.0724 to 0.0116)	0.0214	–0.0476 (–0.0963 to 0.0011)	0.0249	–0.0460 (–0.0950 to 0.0029)	0.0250
4-8 y						
Year of surgery	–0.0328 (–0.0759 to 0.0103)	0.0219	–0.0291 (–0.0887 to 0.0305)	0.0304	–0.0178 (–0.0689 to 0.0333)	0.0261
Mean follow-up	–0.3871 (–0.6656 to –0.1085)	0.1421	0.0426 (–0.2941 to 0.3793)	0.1718	–0.2489 (–0.5925 to 0.0929)	0.1749
Age at surgery	–0.0759 (–0.1281 to –0.0238)	0.0266	0.0135 (–0.0402 to 0.672)	0.0274	–0.0605 (–0.1562 to 0.0352)	0.0488

*^a^* BPTB, bone–patellar tendon–bone.

## Discussion

To the best of our knowledge, this is the first study to investigate revision rates in this way. Our primary aim was to investigate how previous advances in orthopaedics have affected the revision rates after primary ACL reconstruction. Our systematic review and meta-analysis demonstrated an overall pooled revision rate of 3.14% with a median follow-up of 2.3 years. This outlines the fact that ACL reconstruction is a reliable procedure with historically low revision rates. Although we found that the revision rates increased between surgeries performed with hamstring autograft, the most used graft in ACL reconstruction, in 1969 and 2018^34^ (β = 0.0434; 95% CI, 0.0150-0.0718), the overall revision rate was still very low (2.71%; 95% CI, 2.25%-3.27%) for a common orthopaedic procedure.

Previous meta-analyses have reported revision rates ranging from 2.80% to 7%.^5,25,28,32^ At the patient level, the risk of failure is affected by multiple factors, such as graft type, age, and body mass index.^8,25,30,32^ Graft type has been a widely investigated topic, as the revision rate is associated with graft choice.^3,8,20,23,25^ BPTB is reported to have the lowest revision rate.^3,8,22,23^ The risk for graft rupture and/or revision surgery after BPTB reconstruction has been reported to be between 1.3% and 4.0% in the 5-year follow-up period.^8,14,18,21^ For reconstruction with hamstrings, these risks have been reported to be between 2.7% and 15.0% in the same period.^14,18,21^ In this study, patients who received BPTB autograft in primary reconstruction had a 2.38% risk for revision surgery, with a median follow-up of 2.2 years. For hamstring autografts, we observed a 2.71% revision rate, with a median follow-up of 2.1 years, which is slightly inferior to BPTB. From the perspective of the individual patient, the minor difference in the average risk for revision surgery between the BPTB and hamstring might not be crucial when deciding the treatment of the torn ACL for an optimal result.^17^

Failure after primary ACL reconstruction is a very important factor for an individual patient. Based on the previously published literature, as well as our analysis, ACL reconstruction with BPTB graft has very low reported revision rates through the history of ACL reconstruction.^3,8,22,23^ For hamstring grafts, the revision rates have increased, but are very low for a common orthopaedic procedure, with excellent patient-reported outcomes. It is a positive finding that revision rates have remained very low throughout the history of ACL reconstruction, which makes it a reliable procedure for everyday practice. By choosing the age at the time of surgery and follow-up time as other confounding factors besides the operational year, we successfully investigated the effect of the operational year on the observed revision rates. We also found a positive correlation between patient age at surgery and decreased revision rates, which has been previously reported in the literature.^6,12^ We did not conduct subgroup analyses based on sex, as the current consensus seems to be that different revision rates are not affected by sex.^12,24^ Overall, both BPTB and hamstring can be considered reliable graft choices for ACL reconstruction.

A 2018 study published by Zbrojkiewicz et al^33^ found that from 2000 to 2015, the annual incidence of ACL reconstruction increased by 43%, from 54.0 to 77.4 per 100,000 in the Australian population. However, the overall incidence of ACL revisions has increased by 127%, or by 5.6% per year. Hence, the incidence of revision surgery has increased more than the incidence of primary reconstructions. With the increased knowledge and development of new surgical techniques, this might be considered a surprising finding. Moreover, with the increased amount of literature on ligamentous knee injuries, we might assume that the surgical techniques have also evolved in revision surgeries, and therefore we are able to reoperate on more patients than in the beginning of the 21st century. Also, the increased knowledge on the sources of failure might have changed our indication for reoperation. Our analysis showed that revision rates have remained low with no upward trend. Especially the revision rates after primary reconstruction with BPTB have been low throughout the history of ACL surgery. The strongest and most significant effect between operation years and revision rates was after reconstructions with the hamstring as a graft, where 1 year effected a 0.0434 increase in the risk of revision surgery on a logit-transformed scale. But still, the revision rates for hamstring graft were very close to those observed in the BPTB group, and the increasing trend of the revision rates might be partly explained by the changing indications on the revision surgeries. Due to the systematic review and meta-analysis, our study had a more heterogeneous cohort, as compared with that of Zbrojkiewicz et al; with a sample size of almost 50,000 patients, we managed to produce unique information on the progress of the revision rates.

Our findings might also be partly explained by the changing indications for surgical treatment of primary ACL ruptures. Currently, with increasing knowledge and development of surgical techniques, we might choose to operate on more risky patients, which might not have been operated on some time earlier. A 2010 RCT by Frobell et al^4^ reported that for a patient with an acute ACL tear, rehabilitation, combined with an optional delayed reconstruction, results in similar outcomes in the 2-year period, as compared with the early ACL reconstruction surgery. During the 2-year follow-up, only 23 (39%) of these patients underwent delayed reconstruction due to self-reported symptomatic instability caused by insufficient ACL and positive pivot shift. In patients with an early ACL reconstruction, the results were slightly inferior compared with those of the patients with rehabilitation only or with those of the patients who had undergone delayed ACL reconstruction. If nonoperative treatment results in similar or even superior outcomes than early reconstruction, it might be possible that the demographics of the patients who are treated surgically have changed and the preoperative risk for revision is greater than it has been before. This may have affected the observed failure rates.

ACL surgery has many surgical variables that must be optimized based on the estimated revision rate or risk for any other complications. However, patients and surgeons might not share the same priorities related to ACL reconstruction and treatment decisions.^17^ When planning future research, it is necessary to evaluate the most crucial questions. As the number of previously published studies is massive, we can truly ask how much more research is needed, for example, on graft choice and graft fixation techniques. We postulate that we should focus more on patient-specific differences on the ACL surgery, and how they affect the outcome after surgically treated ACL injury. In addition to the surgical variables, it is worth focusing on nonoperative treatment, as it has been shown to result in outcomes similar to those of early reconstruction.^4^

### Strengths and Limitations

Our study has several limitations. The year of the operation was calculated as the mean based on the operational years, which might not be the exact truth. However, with our sample size of more than 300 studies, differences in the exact time of operation may not have been that significant. The same method was used for every study included in our analysis. Another limitation of our study was that not all studies reported the exact reason for failure. For example, the definition of graft failure may include graft ruptures. We have reported the definitions of failure as did the original author from the analyzed article. The third limitation of our study is that in some studies, primary ACL reconstruction failed, but not all failures were revised. We included the number of revision surgeries performed in our analysis. Finally, the choice to undergo revision surgery is based on a single patient’s preferences. However, our aim was to investigate revision rates rather than failure rates. The fourth and main limitation in our study is a rather heterogenic categorization between graft origins. However, we do believe that our results simplify how ACL surgery has developed throughout our 45-year study period. In particular, the “other” group includes very different graft types, but as stated before, we believe that this categorization simplifies how the previous advances in orthopaedics have affected the revision rates in ACL surgery.

The strength of this study is the sample size; thus, the number of not-revised failures is not that significant. It would have barely produced any difference in our results.

## Conclusion

Based on our systematic review and meta-analysis, ACL reconstruction is a reliable procedure with overall historically low revision rates. Our meta-analysis revealed that revision rates are comparable with those of the most used graft types, with hamstrings slightly inferior to BPTB. Overall, both BPTB and hamstring autografts are reliable graft choices for ACL reconstruction.

Supplemental Material for this article is available at http://journals.sagepub.com/doi/suppl/10.1177/23259671221110191

## Supplemental Material

Supplemental Material, sj-pdf-1-ojs-10.1177_23259671221110191 - Revision Rates After Primary ACL Reconstruction Performed Between 1969 and 2018: A Systematic Review and Metaregression Analysis Click here for additional data file. Supplemental Material, sj-pdf-1-ojs-10.1177_23259671221110191 for Revision Rates After Primary ACL Reconstruction Performed Between 1969 and 2018: A Systematic Review and Metaregression Analysis by Rasmus J. Liukkonen, Ville T. Ponkilainen and Aleksi Reito in Orthopaedic Journal of Sports Medicine

## References

[1] Ardern CL Webster KE Taylor NF Feller JA . Return to sport following anterior cruciate ligament reconstruction surgery: a systematic review and meta-analysis of the state of play. Br J Sports Med. 2011;45(7):596–606. 21398310 10.1136/bjsm.2010.076364

[2] Chambat P Guier C Sonnery-Cottet B Fayard JM Thaunat M . The evolution of ACL reconstruction over the last fifty years. Int Orthop. 2013;37(2):181–186. 23322063 10.1007/s00264-012-1759-3 PMC3560904

[3] Ekeland A Engebretsen L Fenstad AM Heir S . Similar risk of ACL graft revision for alpine skiers, football and handball players: the graft revision rate is influenced by age and graft choice. Br J Sports Med. 2020;54(1):33–37. 31399428 10.1136/bjsports-2018-100020

[4] Frobell RB Roos EM Roos HP Ranstam J Lohmander LS . A randomized trial of treatment for acute anterior cruciate ligament tears. N Engl J Med. 2010;363(4):331–342. 20660401 10.1056/NEJMoa0907797

[5] Gabler CM Jacobs CA Howard JS Mattacola CG Johnson DL . Comparison of graft failure rate between autografts placed via an anatomic anterior cruciate ligament reconstruction technique: a systematic review, meta-analysis, and meta-regression. Am J Sports Med. 2016;44(4):1069–1079. 25999439 10.1177/0363546515584043

[6] Gallo MC Bolia IK Jalali O , et al. Risk factors for early subsequent (revision or contralateral) ACL reconstruction: a retrospective database study. Orthop J Sports Med. 2020;8(2):2325967119901173. 32118083 10.1177/2325967119901173 PMC7029539

[7] Garrett WEJ Swiontkowski MF Weinstein JN , et al. American Board of Orthopaedic Surgery practice of the orthopaedic surgeon: part-II, certification examination case mix. J Bone Joint Surg Am. 2006;88(3):660–667. 16510834 10.2106/JBJS.E.01208

[8] Gifstad T Foss OA Engebretsen L , et al. Lower risk of revision with patellar tendon autografts compared with hamstring autografts: a registry study based on 45,998 primary ACL reconstructions in Scandinavia. Am J Sports Med. 2014;42(10):2319–2328. 25201444 10.1177/0363546514548164

[9] Harilainen A Sandelin J. Revision anterior cruciate ligament surgery. A review of the literature and results of our own revisions. Scand J Med Sci Sports. 2001;11(3):163–169. 11374430

[10] Howell SM Taylor Ma . Failure of reconstruction of the anterior cruciate ligament due to impingement by the intercondylar roof. J Bone Joint Surg Am. 1993;75(7):1044–1055. 8335664 10.2106/00004623-199307000-00011

[11] Johnson RJ Eriksson E Haggmark T Pope MH . Five- to ten-year follow-up evaluation after reconstruction of the anterior cruciate ligament. Clin Orthop Relat Res. 1984;183:122–140. 6365386

[12] Kaeding CC Pedroza AD Reinke EK Huston LJ ; MOON Consortium; Spindler KP. Risk factors and predictors of subsequent ACL injury in either knee after ACL reconstruction: prospective analysis of 2488 primary ACL reconstructions from the MOON cohort. Am J Sports Med. 2015;43(7):1583–1590. 25899429 10.1177/0363546515578836 PMC4601557

[13] Kay J Memon M Sa Dd , et al. A historical analysis of randomized controlled trials in anterior cruciate ligament surgery. J Bone Joint Surg Am. 2017;99(24):2062–2068. 29257011 10.2106/JBJS.16.01408

[14] Laboute E James-Belin E Puig PL Trouve P Verhaeghe E . Graft failure is more frequent after hamstring than patellar tendon autograft. Knee Surg Sports Traumatol Arthrosc. 2018;26(12):3537–3546. 29767271 10.1007/s00167-018-4982-7

[15] Mall NA Chalmers PN Moric M , et al. Incidence and trends of anterior cruciate ligament reconstruction in the United States. Am J Sports Med. 2014;42(10):2363–2370. 25086064 10.1177/0363546514542796

[16] Markatos K Kaseta MK Lallos SN Korres DS Efstathopoulos N . The anatomy of the ACL and its importance in ACL reconstruction. Eur J Orthop Surg Traumatol. 2013;23(7):747–752. 23412211 10.1007/s00590-012-1079-8

[17] Marmura H Bryant DM Birmingham TB , et al. Same knee, different goals: patients and surgeons have different priorities related to ACL reconstruction. Knee Surg Sports Traumatol Arthrosc. 2021;29(12):4286–4295. 33876273 10.1007/s00167-021-06550-7

[18] Mohtadi NG Chan DS . A randomized clinical trial comparing patellar tendon, hamstring tendon, and double-bundle ACL reconstructions: patient-reported and clinical outcomes at 5-year follow-up. J Bone Joint Surg Am. 2019;101(11):949–960. 31169571 10.2106/JBJS.18.01322

[19] Musahl V Karlsson J . Anterior cruciate ligament tear. N Engl J Med. 2019;380(24):2341–2348. 31189037 10.1056/NEJMcp1805931

[20] Persson A Fjeldsgaard K Gjertsen J , et al. Increased risk of revision with hamstring tendon grafts compared with patellar tendon grafts after anterior cruciate ligament reconstruction: a study of 12,643 patients from the Norwegian Cruciate Ligament Registry, 2004-2012. Am J Sports Med. 2014;42(2):285–291. 24322979 10.1177/0363546513511419

[21] Rahardja R Zhu M Love H Clatworthy MG Monk AP Young SW . Effect of graft choice on revision and contralateral anterior cruciate ligament reconstruction: results from the New Zealand ACL Registry. Am J Sports Med. 2020;48(1):63–69. 31730379 10.1177/0363546519885148

[22] Rahardja R Zhu M Love H Clatworthy MG Monk AP Young SW . Rates of revision and surgeon-reported graft rupture following ACL reconstruction: early results from the New Zealand ACL Registry. Knee Surg Sports Traumatol Arthrosc. 2020;28(7):2194–2202. 31679071 10.1007/s00167-019-05773-z

[23] Rousseau R Labruyere C Kajetanek C Deschamps O Makridis KG Djian P . Complications after anterior cruciate ligament reconstruction and their relation to the type of graft: a prospective study of 958 cases. Am J Sports Med. 2019;47(11):2543–2549. 31403824 10.1177/0363546519867913

[24] Ryan J Magnussen RA Cox CL Hurbanek JG Flanigan DC Kaeding CC . ACL reconstruction: do outcomes differ by sex? A systematic review. J Bone Joint Surg Am. 2014;96(6):507–512. 24647508 10.2106/JBJS.M.00299

[25] Samuelsen BT Webster KE Johnson NR Hewett TE Krych AJ . Hamstring autograft versus patellar tendon autograft for ACL reconstruction: is there a difference in graft failure rate? A meta-analysis of 47,613 patients. Clin Orthop Relat Res. 2017;475(10):2459–2468. 28205075 10.1007/s11999-017-5278-9 PMC5599382

[26] Satora W Królikowska A Czamara A Reichert P . Synthetic grafts in the treatment of ruptured anterior cruciate ligament of the knee joint. Polim Med. 2017;47(1):55–59. 29160630 10.17219/pim/76819

[27] Schindler OS . Surgery for anterior cruciate ligament deficiency: a historical perspective. Knee Surg Sports Traumatol Arthrosc. 2012;20(1):5–47. 22105976 10.1007/s00167-011-1756-x

[28] Sepúlveda F Sánchez L Amy E Micheo W . Anterior cruciate ligament injury: return to play, function and long-term considerations. Curr Sports Med Rep. 2017;16(3):172–178. 28498226 10.1249/JSR.0000000000000356

[29] Siegel L Vandenakker-Albanese C Siegel D . Anterior cruciate ligament injuries: anatomy, physiology, biomechanics, and management. Clin J Sport Med. 2012;22(4):349–355. 22695402 10.1097/JSM.0b013e3182580cd0

[30] Traven SA Reeves RA Xerogeanes JW Slone HS . Higher BMI predicts additional surgery at the time of ACL reconstruction. Knee Surg Sports Traumatol Arthrosc. 2019;27(8):2552–2557. 30374577 10.1007/s00167-018-5267-x

[31] van Melick N van Cingel RE Brooijmans F , et al. Evidence-based clinical practice update: practice guidelines for anterior cruciate ligament rehabilitation based on a systematic review and multidisciplinary consensus. Br J Sports Med. 2016;50(24):1506–1515. 27539507 10.1136/bjsports-2015-095898

[32] Wiggins AJ Grandhi RK Schneider DK Stanfield D Webster KE Myer GD . Risk of secondary injury in younger athletes after anterior cruciate ligament reconstruction. Am J Sports Med. 2016;44(7):1861–1876. 26772611 10.1177/0363546515621554 PMC5501245

[33] Zbrojkiewicz D Vertullo C Grayson JE . Increasing rates of anterior cruciate ligament reconstruction in young Australians, 2000–2015. Med J Aust. 2018;208(8):354–358. 29669497 10.5694/mja17.00974

[34] Ziegler CG DePhillipo NN Kennedy MI Dekker TJ Dornan GJ LaPrade RF . Beighton Score, Tibial slope, tibial subluxation, quadriceps circumference difference, and family history are risk factors for anterior cruciate ligament graft failure: A retrospective comparison of primary and revision anterior cruciate ligament reconstructions. Arthroscopy. 2021;37(1):195–205. 32911007 10.1016/j.arthro.2020.08.031

