# Supplementary material for: Revision Rates After Primary ACL Reconstruction Performed Between 1969 and 2018: A Systematic Review and Metaregression Analysis
Source: Orthop J Sports Med. 2022 Aug 5;10(8):23259671221110191. doi: 10.1177/23259671221110191 (PMC9358584; doi:10.1177/23259671221110191)
Supplement: Supplemental Material, sj-pdf-1-ojs-10.1177_23259671221110191 - Revision Rates After Primary ACL Reconstruction Performed Between 1969 and 2018: A Systematic Review and Metaregression Analysis [file sj-pdf-1-ojs-10.1177_23259671221110191.pdf]

Supplemental Material: Reference List of Articles Included in This Review

1. Abdul W, Guro R, Jawad Z, Kotwal R, Chandratreya A. Clinical outcomes of primary anatomic all-inside anterior cruciate ligament reconstruction using the translateral technique: a minimum one-year follow-up study. *Knee.* 2020;27:1753-1763.

2. Adachi N, Ochi M, Uchio Y, Iwasa J, Kuriwaka M, Ito Y. Reconstruction of the anterior cruciate ligament. Single- versus double-bundle multistranded hamstring tendons. *J Bone Joint Surg Br.* 2004;86:515-520.

3. Aglietti P, Buzzi R, Menchetti PM, Giron F. Arthroscopically assisted semitendinosus and gracilis tendon graft in reconstruction for acute anterior cruciate ligament injuries in athletes. *Am J Sports Med.* 1996;24:726-731.

4. Aglietti P, Giron F, Buzzi R, Biddau F, Sasso F. Anterior cruciate ligament reconstruction: bone-patellar tendon-bone compared with double semitendinosus and gracilis tendon grafts. A prospective, randomized clinical trial. *J Bone Joint Surg Am.* 2004;86:2143-2155.

5. Aglietti P, Giron F, Losco M, Cuomo P, Ciardullo A, Mondanelli N. Comparison between single-and double-bundle anterior cruciate ligament reconstruction: a prospective, randomized, single-blinded clinical trial. *Am J Sports Med.* 2010;38:25-34.

6. Ahldén M, Sernert N, Karlsson J, Kartus J. A prospective randomized study comparing double- and single-bundle techniques for anterior cruciate ligament reconstruction. *Am J Sports Med.* 2013;41:2484-2491.

7. Ahn JH, Kim J, Mun JW. A retrospective comparison of single-bundle anterior cruciate ligament reconstruction with lateral extra-articular tenodesis with double-bundle anterior cruciate ligament reconstruction. *Arthroscopy.* 2021;37:976-984.

8. Ahn JH, Park JS, Lee YS, Cho YJ. Femoral bioabsorbable cross-pin fixation in anterior cruciate ligament reconstruction. *Arthroscopy.* 2007;23:1093-1099.

9. Allahabadi S, Rubenstein WJ, Lansdown DA, Feeley BT, Pandya NK. Incidence of anterior cruciate ligament graft tears in high-risk populations: An analysis of professional athlete and pediatric populations. *Knee.* 2020;27:1378-1384.

10. Anderson AF, Snyder RB, Lipscomb AB Jr. Anterior cruciate ligament reconstruction. A prospective randomized study of three surgical methods. *Am J Sports Med.* 2001;29:272-279.

11. Andersson C, Odensten M, Gillquist J. Knee function after surgical or nonsurgical treatment of acute rupture of the anterior cruciate ligament: a randomized study with a long-term follow-up period. *Clin Orthop Relat Res.* 1991;264:255-263.

12. Annear PT, Rohr EJ, Hille DM, Gohil S, Ebert JR. No clinical difference in 10-year outcomes between standard and minimal graft debridement techniques in patients undergoing anterior cruciate ligament reconstruction using autologous hamstrings: a randomized controlled trial. *Knee Surg Sports Traumatol Arthrosc.* 2019;27:516-523.

13. Antosh IJ, Patzkowski JC, Racusin AW, Aden JK, Waterman SM. Return to military duty after anterior cruciate ligament reconstruction. *Mil Med.* 2018;183:e83-e89.

14. Araki D, Kuroda R, Kubo S, et al. A prospective randomised study of anatomical single-bundle versus double-bundle anterior cruciate ligament reconstruction: quantitative evaluation using an electromagnetic measurement system. *Int Orthop.* 2011;35:439-446.

15. Arama Y, Salmon LJ, Sri-Ram K, Linklater J, Roe JP, Pinczewski LA. Bioabsorbable versus titanium screws in anterior cruciate ligament reconstruction using hamstring autograft: a prospective, blinded, randomized controlled trial with 5-year follow-up. *Am J Sports Med.* 2015;43:1893-1901.

16. Arneja S, Froese W, MacDonald P. Augmentation of femoral fixation in hamstring anterior cruciate ligament reconstruction with a bioabsorbable bead: a prospective single-blind randomized clinical trial. *Am J Sports Med.* 2004;32:159-163.

17. Attia AK, Nasef H, ElSweify KH, Adam MA, AbuShaaban F, Arun K. Failure rates of 5-strand and 6-strand vs quadrupled hamstring autograft ACL reconstruction: a comparative study of 413 patients with a minimum 2-year follow-up. *Orthop J Sports Med.* 2020;8:2325967120946326.

18. Bach BR Jr, Tradonsky S, Bojchuk J, Levy ME, Bush-Joseph CA, Khan NH. Arthroscopically assisted anterior cruciate ligament reconstruction using patellar tendon autograft. Five- to nine-year follow-up evaluation. *Am J Sports Med.* 1998;26:20-29.

19. Bak K, Jørgensen U, Ekstrand J, Scavenius M. Reconstruction of anterior cruciate ligament deficient knees in soccer players with an iliotibial band autograft. A prospective study of 132 reconstructed knees followed for 4 (2-7) years. *Scand J Med Sci Sports.* 2001;11:16-22.

20. Bak K, Jørgensen U, Ekstrand J, Scavenius M. Results of reconstruction of acute ruptures of the anterior cruciate ligament with an iliotibial band autograft. *Knee Surg Sports Traumatol Arthrosc.* 1997;7:111-117.

21. Bangert Y, Jaber A, Wünnemann F, et al. Clinical and radiological outcome after anterior cruciate ligament reconstruction using the T-lock Osteotrans resorbable tendon anchor: early experience and midterm follow-up. *BMC Musculoskelet Disord.* 2020;21:844.

22. Barber FA, Boothby MH. Bilok interference screws for anterior cruciate ligament reconstruction: clinical and radiographic outcomes. *Arthroscopy.* 2007;23:476-481.

23. Barber FA, Cowden CH 3rd, Sanders EJ. Revision rates after anterior cruciate ligament reconstruction using bone-patellar tendon-bone allograft or autograft in a population 25 years old and younger. *Arthroscopy.* 2014;30:483-491.

24. Barber-Westin SD, Noyes FR, Andrews M. A rigorous comparison between the sexes of results and complications after anterior cruciate ligament reconstruction. *Am J Sports Med.* 1997;25:514-526.

25. Barié A, Sprinckstub T, Huber J, Jaber A. Quadriceps tendon vs. patellar tendon autograft for ACL reconstruction using a hardware-free press-fit fixation technique: comparable stability, function and return-to-sport level but less donor site morbidity in athletes after 10 years. *Arch Orthop Trauma Surg.* 2020;140:1465-1474.

26. Barrett AM, Craft JA, Replogle WH, Hydrick JM, Barrett GR. Anterior cruciate ligament graft failure: a comparison of graft type based on age and Tegner activity level. *Am J Sports Med.* 2011;39:2194-2198.

27. Barrett G, Stokes D, White M. Anterior cruciate ligament reconstruction in patients older than 40 years: allograft versus autograft patellar tendon. *Am J Sports Med.* 2005;33:1505-1512.

28. Barrett GR, Line LL Jr, Shelton WR, Manning JO, Phelps R. The Dacron ligament prosthesis in anterior cruciate ligament reconstruction. A four-year review. *Am J Sports Med.* 1993;21:367-373.

29. Barrett GR, Luber K, Replogle WH, Manley JL. Allograft anterior cruciate ligament reconstruction in the young, active patient: Tegner activity level and failure rate. *Arthroscopy.* 2010;26:1593-1601.

30. Barrett GR, Noojin FK, Hartzog CW, Nash CR. Reconstruction of the anterior cruciate ligament in females: A comparison of hamstring versus patellar tendon autograft. *Arthroscopy.* 2002;18:46-54.

31. Barrett GR, Treacy SH. The effect of intraoperative isometric measurement on the outcome of anterior cruciate ligament reconstruction: a clinical analysis. *Arthroscopy.* 1996;12:645-651.

32. Beard DJ, Anderson JL, Davies S, Price AJ, Dodd CA. Hamstrings vs. patella tendon for anterior cruciate ligament reconstruction: a randomised controlled trial. *Knee.* 2001;8:45-50.

33. Benedetto KP, Fellinger M, Lim TE, Passler JM, Schoen JL, Willems WJ. A new bioabsorbable interference screw: preliminary results of a prospective, multicenter, randomized clinical trial. *Arthroscopy.* 2000;16:41-48.

34. Benner RW, Shelbourne KD, Gray T. The degree of knee extension does not affect postoperative stability or subsequent graft tear rate after anterior cruciate ligament reconstruction with patellar tendon autograft. *Am J Sports Med.* 2016;44:844-849.

35. Berdis AS, Veale K, Fleissner PR Jr. Outcomes of anterior cruciate ligament reconstruction using biologic augmentation in patients 21 years of age and younger. *Arthroscopy.* 2019;35:3107-3113.

36. Beynnon BD, Johnson RJ, Fleming BC, et al. Anterior cruciate ligament replacement: comparison of bone-patellar tendon-bone grafts with two-strand hamstring grafts. A prospective, randomized study. *J Bone Joint Surg Am.* 2002;84:1503-1513.

37. Beynnon BD, Johnson RJ, Naud S, et al. Accelerated versus nonaccelerated rehabilitation after anterior cruciate ligament reconstruction: a prospective, randomized, double-blind investigation evaluating knee joint laxity using roentgen stereophotogrammetric analysis. *Am J Sports Med.* 2011;39:2536-2548.

38. Beynnon BD, Uh BS, Johnson RJ, et al. Rehabilitation after anterior cruciate ligament reconstruction: a prospective, randomized, double-blind comparison of programs administered over 2 different time intervals. *Am J Sports Med.* 2005;33:347-359.

39. Bin Abd Razak HR, Chong HC, Tan HA. Obesity is associated with poorer range of motion and Tegner scores following hamstring autograft anterior cruciate ligament reconstruction in Asians. *Ann Transl Med.* 2017;5:304.

40. Birmingham TB, Bryant DM, Giffin JR, et al. A randomized controlled trial comparing the effectiveness of functional knee brace and neoprene sleeve use after anterior cruciate ligament reconstruction. *Am J Sports Med.* 2008;36:648-655.

41. Blyth MJ, Gosal HS, Peake WM, Bartlett RJ. Anterior cruciate ligament reconstruction in patients over the age of 50 years: 2- to 8-year follow-up. *Knee Surg Sports Traumatol Arthrosc.* 2003;11:204-11.

42. Bohn MB, Sørensen H, Petersen MK, Søballe K, Lind M. Rotational laxity after anatomical ACL reconstruction measured by 3-D motion analysis: a prospective randomized clinical trial comparing anatomic and nonanatomic ACL reconstruction techniques. *Knee Surg Sports Traumatol Arthrosc.* 2015;23:3473-3481.

43. Borton ZM, Yasen SK, Mumith A, Wilson AJ. Mid-bundle positioning of the femoral socket increases graft rupture in anatomic single bundle anterior cruciate ligament reconstruction. *Knee.* 2018;25:1122-1128.

44. Boszotta H. Arthroscopic anterior cruciate ligament reconstruction using a patellar tendon graft in press-fit technique: surgical technique and follow-up. *Arthroscopy.* 1997;13:332-339.

45. Bottoni CR, Liddell TR, Trainor TJ, Freccero DM, Lindell KK. Postoperative range of motion following anterior cruciate ligament reconstruction using autograft hamstrings: a prospective, randomized clinical trial of early versus delayed reconstructions. *Am J Sports Med.* 2008;36:656-662.

46. Bourke HE, Salmon LJ, Waller A, et al. Randomized controlled trial of osteoconductive fixation screws for anterior cruciate ligament reconstruction: a comparison of the Calaxo and Milagro screws. *Arthroscopy.* 2013;29:74-82.

47. Brandsson S, Faxén E, Eriksson BI, et al. Closing patellar tendon defects after anterior cruciate ligament reconstruction: absence of any benefit. *Knee Surg Sports Traumatol Arthrosc.* 1998;6:82-87.

48. Brandsson S, Faxén E, Kartus J, Eriksson BI, Karlsson J. Is a knee brace advantageous after anterior cruciate ligament surgery? A prospective, randomised study with a two-year follow-up. *Scand J Med Sci Sports.* 2001;11:110-114.

49. Britt E, Ouillette R, Edmonds E, et al. The challenges of treating female soccer players with ACL injuries: hamstring versus bone-patellar tendon-bone autograft. *Orthop J Sports Med.* 2020;8:2325967120964884.

50. Buchner M, Schmeer T, Schmitt H. Anterior cruciate ligament reconstruction with quadrupled semitendinosus tendon - minimum 6 year clinical and radiological follow-up. *Knee.* 2007;14:321-327.

51. Buda R, Baldassarri M, Perazzo L, Ghinelli D, Faldini C. The biological respect of the posterolateral bundle in ACL partial injuries. Retrospective analysis of 2 different surgical management of ACL partial tear in a population of high-demanding sport patients. *Eur J Orthop Surg Traumatol.* 2019;29:651-658.

52. Burrus MT, Werner BC, Crow AJ, et al. Increased failure rates after anterior cruciate ligament reconstruction with soft-tissue autograft-allograft hybrid grafts. *Arthroscopy.* 2015;31:2342-2351.

53. Campbell AC, Rae PS. Anterior cruciate reconstruction with the ABC carbon and polyester prosthetic ligament. *Ann R Coll Surg Engl.* 1995;77:349-350.

54. CarlLee T, Ries Z, Duchman K, et al. Outside-in vs. anteromedial portal drilling during primary ACL reconstruction: comparison at two years. *Iowa Orthop J.* 2017;37:117-122.

55. Carulli C, Matassi F, Soderi S, Sirleo L, Munz G, Innocenti M. Resorbable screw and sheath versus resorbable interference screw and staples for ACL reconstruction: a comparison of two tibial fixation methods. *Knee Surg Sports Traumatol Arthrosc.* 2017;25:1264-1271.

56. Chen J, Gu A, Jiang H, Zhang W, Yu X. A comparison of acute and chronic anterior cruciate ligament reconstruction using LARS artificial ligaments: a randomized prospective study with a 5-year follow-up. *Arch Orthop Trauma Surg.* 2015;135:95-102.

57. Colombet P, Saffarini M, Bouguennec N. Clinical and functional outcomes of anterior cruciate ligament reconstruction at a minimum of 2 years using adjustable suspensory fixation in both the femur and tibia: a prospective study. *Orthop J Sports Med.* 2018;6:2325967118804128.

58. Cooley VJ, Deffner KT, Rosenberg TD. Quadrupled semitendinosus anterior cruciate ligament reconstruction: 5-year results in patients without meniscus loss. *Arthroscopy.* 2001;17:795-800.

59. Cordasco FA, Black SR, Price M, et al. Return to sport and reoperation rates in patients under the age of 20 after primary anterior cruciate ligament reconstruction: risk profile comparing 3 patient groups predicated upon skeletal age. *Am J Sports Med.* 2019;47:628-639.

60. Cruz CA, Goldberg D, Wake J, et al. Comparing bone-tendon autograft with bone-tendon-bone autograft for ACL reconstruction: a matched-cohort analysis. *Orthop J Sports Med.* 2020;8:2325967120970224.

61. D'Alessandro P, Wake G, Annear P. Hamstring pain and muscle strains following anterior cruciate ligament reconstruction: a prospective, randomized trial comparing hamstring graft harvest techniques. *J Knee Surg.* 2013;26:139-144.

62. da Silva Guarilha E, de Andrade Fígaro Caldeira PR, de Almeida Lira Neto O, Navarro MS, Milani A, Filho MC. Randomized prospective study comparing transverse and extracortical fixation in anterior cruciate ligament reconstruction. *Rev Bras Ortop.* 2015;47:354-358.

63. Dahlstedt L, Dalén N, Jonsson U. Goretex prosthetic ligament vs. Kennedy ligament augmentation device in anterior cruciate ligament reconstruction. A prospective randomized 3-year follow-up of 41 cases. *Acta Orthop Scand.* 1990;61:217-224.

64. Dahlstedt LJ, Netz P, Dalén N. Poor results of bovine xenograft for knee cruciate ligament repair. *Acta Orthop Scand.* 1989;60:3-7.

65. Dahm DL, Wulf CA, Dajani KA, Dobbs RE, Levy BA, Stuart MA. Reconstruction of the anterior cruciate ligament in patients over 50 years. *J Bone Joint Surg Br.* 2008;90:1446-1450.

66. Dai C, Wang F, Wang X, Wang R, Wang S, Tang S. Arthroscopic single-bundle anterior cruciate ligament reconstruction with six-strand hamstring tendon allograft versus bone-patellar tendon-bone allograft. *Knee Surg Sports Traumatol Arthrosc.* 2016;24:2915-2922.

67. Darnley JE, Léger-St-Jean B, Pedroza AD, Flanigan DC, Kaeding CC, Magnussen RA. Anterior cruciate ligament reconstruction using a combination of autograft and allograft tendon: a MOON cohort study. *Orthop J Sports Med.* 2016;4:2325967116662249.

68. de Paula Leite Cury R, Simabukuro AM, de Marques Oliveira V, et al. Anteromedial positioning of the femoral tunnel in anterior cruciate ligament reconstruction is the best option to avoid revision: a single surgeon registry. *J Exp Orthop.* 2020;7:11.

69. De Wall M, Scholes CJ, Patel S, Coolican MR, Parker DA. Tibial fixation in anterior cruciate ligament reconstruction: a prospective randomized study comparing metal interference screw and staples with a centrally placed polyethylene screw and sheath. *Am J Sports Med.* 2011;39:1858-1864.

70. Deehan DJ, Salmon LJ, Webb VJ, Davies A, Pinczewski LA. Endoscopic reconstruction of the anterior cruciate ligament with an ipsilateral patellar tendon autograft. A prospective longitudinal five-year study. *J Bone Joint Surg Br.* 2000;82:984-991.

71. DeFrancesco CJ, Storey EP, Flynn JM, Ganley TJ. Pediatric ACL reconstruction and return to the operating room: revision is less than half of the story. *J Pediatr Orthop.* 2019;39:516-520.

72. Denti M, Bigoni M, Dodaro G, Monteleone M, Arosio A. Long-term results of the Leeds-Keio anterior cruciate ligament reconstruction. *Knee Surg Sports Traumatol Arthrosc.* 1995;3:75-77.

73. Desai VS, Anderson GR, Wu IT, et al. Anterior cruciate ligament reconstruction with hamstring autograft: a matched cohort comparison of the all-inside and complete tibial tunnel techniques. *Orthop J Sports Med.* 2019;7:2325967118820297.

74. Diermeier T, Meredith SJ, Irrgang JJ, et al. Patient-reported and quantitative outcomes of anatomic anterior cruciate ligament reconstruction with hamstring tendon autografts. *Orthop J Sports Med.* 2020;8:2325967120926159.

75. Drogset JO, Grøntvedt T. Anterior cruciate ligament reconstruction with and without a ligament augmentation device: results at 8-Year follow-up. *Am J Sports Med.* 2002;30:851-856.

76. Drogset JO, Strand T, Uppheim G, Odegård B, Bøe A, Grøntvedt T. Autologous patellar tendon and quadrupled hamstring grafts in anterior cruciate ligament reconstruction: a prospective randomized multicenter review of different fixation methods. *Knee Surg Sports Traumatol Arthrosc.* 2010;18:1085-1093.

77. Drogset JO, Straume LG, Bjørkmo I, Myhr G. A prospective randomized study of ACL-reconstructions using bone-patellar tendon-bone grafts fixed with bioabsorbable or metal interference screws. *Knee Surg Sports Traumatol Arthrosc.* 2011;19:753-759.

78. Eajazi A, Madadi F, Madadi F, Boreiri M. Comparison of different methods of femoral fixation anterior cruciate ligament reconstruction. *Acta Med Iran.* 2013;51:444-448.

79. Ebert JR, Annear PT. ACL reconstruction using autologous hamstrings augmented with the ligament augmentation and reconstruction system provides good clinical scores, high levels of satisfaction and return to sport, and a low retear rate at 2 years. *Orthop J Sports Med.* 2019;7:2325967119879079.

80. Edgar CM, Zimmer S, Kakar S, Jones H, Schepsis AA. Prospective comparison of auto and allograft hamstring tendon constructs for ACL reconstruction. *Clin Orthop Relat Res.* 2008;466:2238-2246.

81. Ejerhed L, Kartus J, Köhler K, Sernert N, Brandsson S, Karlsson J. Preconditioning patellar tendon autografts in arthroscopic anterior cruciate ligament reconstruction: a prospective randomized study. *Knee Surg Sports Traumatol Arthrosc.* 2001;9:6-11.

82. Ejerhed L, Kartus J, Sernert N, Köhler K, Karlsson J. Patellar tendon or semitendinosus tendon autografts for anterior cruciate ligament reconstruction? A prospective randomized study with a two-year follow-up. *Am J Sports Med.* 2003;31:19-25.

83. Endele D, Jung C, Becker U, Bauer G, Mauch F. Anterior cruciate ligament reconstruction with and without computer navigation: a clinical and magnetic resonance imaging evaluation 2 years after surgery. *Arthroscopy.* 2009;25:1067-1074.

84. Eriksson K, Anderberg P, Hamberg P, et al. A comparison of quadruple semitendinosus and patellar tendon grafts in reconstruction of the anterior cruciate ligament. *J Bone Joint Surg Br.* 2001;83:348-354.

85. Fauno P, Kaalund S. Tunnel widening after hamstring anterior cruciate ligament reconstruction is influenced by the type of graft fixation used: a prospective randomized study. *Arthroscopy.* 2005;21:1337-1341.

86. Feller JA, Webster KE. A randomized comparison of patellar tendon and hamstring tendon anterior cruciate ligament reconstruction. *Am J Sports Med.* 2003;31:564-573.

87. Ferretti A, Monaco E, Giannetti S, Caperna L, Luzon D, Conteduca F. A medium to long-term follow-up of ACL reconstruction using double gracilis and semitendinosus grafts. *Knee Surg Sports Traumatol Arthrosc.* 2011;19:473-478.

88. Ferretti A, Vadalà A, De Carli A, Argento G, Conteduca F, Severini G. Minimizing internal rotation strength deficit after use of semitendinosus for anterior cruciate ligament reconstruction: a modified harvesting technique. *Arthroscopy.* 2008;24:786-795.

89. Fleming BC, Fadale PD, Hulstyn MJ, et al. The effect of initial graft tension after anterior cruciate ligament reconstruction: a randomized clinical trial with 36-month follow-up. *Am J Sports Med.* 2013;41:25-34.

90. Frobell RB, Roos EM, Roos HP, Ranstam J, Lohmander LS. A randomized trial of treatment for acute anterior cruciate ligament tears. *N Engl J Med.* 2010;363:331-342.

91. Fu FH, Shen W, Starman JS, Okeke N, Irrgang JJ. Primary anatomic double-bundle anterior cruciate ligament reconstruction: a preliminary 2-year prospective study. *Am J Sports Med.* 2008;36:1263-1274.

92. Fujita N, Kuroda R, Matsumoto T, et al. Comparison of the clinical outcome of double-bundle, anteromedial single-bundle, and posterolateral single-bundle anterior cruciate ligament reconstruction using hamstring tendon graft with minimum 2-year follow-up. *Arthroscopy.* 2011;27:906-913.

93. Fukuda TY, Fingerhut D, Moreira VC, et al. Open kinetic chain exercises in a restricted range of motion after anterior cruciate ligament reconstruction: a randomized controlled clinical trial. *Am J Sports Med.* 2013;41:788-794.

94. Funchal LFZ, Astur DC, Ortiz R, Cohen M. The presence of the arthroscopic "floating meniscus" sign as an indicator for surgical intervention in patients with combined anterior cruciate ligament and grade II medial collateral ligament injury. *Arthroscopy.* 2019;35:930-937.

95. Gagliardi AG, Carry PM, Parikh HB, Traver JL, Howell DR, Albright JC. ACL repair with suture ligament augmentation is associated with a high failure rate among adolescent patients. *Am J Sports Med.* 2019;47:560-566.

96. Geib TM, Shelton WR, Phelps RA, Clark L. Anterior cruciate ligament reconstruction using quadriceps tendon autograft: intermediate-term outcome. *Arthroscopy.* 2009;25:1408-1414.

97. Gerich TG, Lattermann C, Fremerey RW, Zeichen J, Lobenhoffer HP. One- versus two-incision technique for anterior cruciate ligament reconstruction with patellar tendon graft. Results on early rehabilitation and stability. *Knee Surg Sports Traumatol Arthrosc.* 1997;5:213-216.

98. Getgood AMJ, Bryant DM, Litchfield R, et al. Lateral extra-articular tenodesis reduces failure of hamstring tendon autograft anterior cruciate ligament reconstruction: 2-year outcomes from the STABILITY Study randomized clinical trial. *Am J Sports Med.* 2020;48:285-297.

99. Ghalayini SR, Helm AT, Bonshahi AY, Lavender A, Johnson DS, Smith RB. Arthroscopic anterior cruciate ligament surgery: results of autogenous patellar tendon graft versus the Leeds-Keio synthetic graft five year follow-up of a prospective randomised controlled trial. *Knee.* 2010;17:334-339.

100. Gifstad T, Drogset JO, Grøntvedt T, Hortemo GS. Femoral fixation of hamstring tendon grafts in ACL reconstructions: the 2-year follow-up results of a prospective randomized controlled study. *Knee Surg Sports Traumatol Arthrosc.* 2014;22:2153-2162.

101. Gobbi A, Domzalski M, Pascual J, Zanazzo M. Hamstring anterior cruciate ligament reconstruction: is it necessary to sacrifice the gracilis? *Arthroscopy.* 2005;21:275-280.

102. Gohil S, Annear PO, Breidahl W. Anterior cruciate ligament reconstruction using autologous double hamstrings: a comparison of standard versus minimal debridement techniques using MRI to assess revascularisation. A randomised prospective study with a one-year follow-up. *J Bone Joint Surg Br.* 2007;89:1165-1171.

103. Gonçalves H, Steltzlen C, Boisrenoult P, Beaufils P, Pujol N. High failure rate of anterior cruciate ligament reconstruction with bimeniscal repair: A case-control study. *Orthop Traumatol Surg Res.* 2017;103:943-946.

104. Grafe MW, Kurzweil PR. Anterior cruciate ligament reconstruction with Achilles tendon allografts in revisions and in patients older than 30. *Am J Orthop (Belle Mead NJ)*. 2008;37:302-308.

105. Grindem H, Snyder-Mackler L, Moksnes H, Engebretsen L, Risberg MA. Simple decision rules can reduce reinjury risk by 84% after ACL reconstruction: the Delaware-Oslo ACL cohort study. *Br J Sports Med.* 2016;50:804-808.

106. Grunau PD, Arneja S, Leith JM. A randomized clinical trial to assess the clinical effectiveness of a measured objective tensioning device in hamstring anterior cruciate ligament reconstruction. *Am J Sports Med.* 2016;44:1482-1486.

107. Gudas R, Jurkonis R, Smailys A. Comparison of return to pre-injury sport after 10 mm size bone-patellar tendon-bone (BPTB) versus 8 mm hamstring anterior cruciate ligament reconstruction: a retrospective study with a two-year follow-up. *Med Sci Monit.* 2018;24:987-996.

108. Guo L, Yang L, Duan XJ, et al. Anterior cruciate ligament reconstruction with bone-patellar tendon-bone graft: comparison of autograft, fresh-frozen allograft, and γ-irradiated allograft. *Arthroscopy*. 2012;28:211-217.

109. Gupta R, Sood M, Malhotra A, et al. Low re-rupture rate with BPTB autograft and semitendinosus gracilis autograft with preserved insertions in ACL reconstruction surgery in sports persons. *Knee Surg Sports Traumatol Arthrosc.* 2018;26:2381-2388.

110. Gürpınar T, Polat B, Eren M, Çarkçı E, Özyalvaç ON, Erdoğan S. The effect of soft tissue interposition of the Endobutton on clinical results and on its postoperative migration after single-bundle anterior cruciate ligament reconstruction. *Knee.* 2020;27:1980-1987.

111. Halinen J, Lindahl J, Hirvensalo E. Range of motion and quadriceps muscle power after early surgical treatment of acute combined anterior cruciate and grade-III medial collateral ligament injuries. A prospective randomized study. *J Bone Joint Surg Am.* 2009;91:1305-1312.

112. Harilainen A, Linko E, Sandelin J. Randomized prospective study of ACL reconstruction with interference screw fixation in patellar tendon autografts versus femoral metal plate suspension and tibial post fixation in hamstring tendon autografts: 5-year clinical and radiological follow-up results. *Knee Surg Sports Traumatol Arthrosc.* 2006;14:517-528.

113. Harilainen A, Sandelin J, Jansson KA. Cross-pin femoral fixation versus metal interference screw fixation in anterior cruciate ligament reconstruction with hamstring tendons: results of a controlled prospective randomized study with 2-year follow-up. *Arthroscopy.* 2005;21:25-33.

114. Harilainen A, Sandelin J, Vanhanen I, Kivinen A. Knee brace after bone-tendon-bone anterior cruciate ligament reconstruction. Randomized, prospective study with 2-year follow-up. *Knee Surg Sports Traumatol Arthrosc.* 1997;5:10-13.

115. Harilainen A, Sandelin J. A prospective comparison of 3 hamstring ACL fixation devices—Rigidfix, BioScrew, and Intrafix—randomized into 4 groups with 2 years of follow-up. *Am J Sports Med.* 2009;37:699-706.

116. Hart R, Krejzla J, Sváb P, Kocis J, Stipcák V. Outcomes after conventional versus computer-navigated anterior cruciate ligament reconstruction. *Arthroscopy.* 2008;24:569-578.

117. Hartigan EH, Axe MJ, Snyder-Mackler L. Time line for noncopers to pass return-to-sports criteria after anterior cruciate ligament reconstruction. *J Orthop Sports Phys Ther.* 2010;40:141-154.

118. Heier KA, Mack DR, Moseley JB, Paine R, Bocell JR. An analysis of anterior cruciate ligament reconstruction in middle-aged patients. *Am J Sports Med.* 1997;25:527-532.

119. Henle P, Bieri KS, Brand M, et al. Patient and surgical characteristics that affect revision risk in dynamic intraligamentary stabilization of the anterior cruciate ligament. *Knee Surg Sports Traumatol Arthrosc.* 2018;26:1182-1189.

120. Henriksson M, Rockborn P, Good L. Range of motion training in brace vs. plaster immobilization after anterior cruciate ligament reconstruction: a prospective randomized comparison with a 2-year follow-up. *Scand J Med Sci Sports.* 2002;12:73-80.

121. Hess T, Duchow J, Roland S, Kohn D. Single-versus two-incision technique in anterior cruciate ligament replacement: influence on postoperative muscle function. *Am J Sports Med.* 2002;30:27-31.

122. Hill PF, Russell VJ, Salmon LJ, Pinczewski LA. The influence of supplementary tibial fixation on laxity measurements after anterior cruciate ligament reconstruction with hamstring tendons in female patients. *Am J Sports Med.* 2005;33:94-101.

123. Ho B, Edmonds EW, Chambers HG, Bastrom TP, Pennock AT. Risk factors for early ACL reconstruction failure in pediatric and adolescent patients: a review of 561 cases. *J Pediatr Orthop* . 2018;38:388-392.

124. Hohmann E, Tetsworth K, Bryant A. Physiotherapy-guided versus home-based, unsupervised rehabilitation in isolated anterior cruciate injuries following surgical reconstruction. *Knee Surg Sports Traumatol Arthrosc.* 2011;19:1158-1167.

125. Holm I, Oiestad BE, Risberg MA, Aune AK. No difference in knee function or prevalence of osteoarthritis after reconstruction of the anterior cruciate ligament with 4-strand hamstring autograft versus patellar tendon-bone autograft: a randomized study with 10-year follow-up. *Am J Sports Med.* 2010;38:448-454.

126. Holmes PF, James SL, Larson RL, Singer KM, Jones DC. Retrospective direct comparison of three intraarticular anterior cruciate ligament reconstructions. *Am J Sports Med.* 1991;19:596-599.

127. Hong L, Li X, Zhang H, et al. Anterior cruciate ligament reconstruction with remnant preservation: a prospective, randomized controlled study. *Am J Sports Med.* 2012;40:2747-2755.

128. Hoogeslag RAG, Brouwer RW, Boer BC, de Vries AJ, Huis In 't Veld R. Acute anterior cruciate ligament rupture: repair or reconstruction? Two-year results of a randomized controlled clinical trial. *Am J Sports Med.* 2019;47:567-577.

129. Hussein M, van Eck CF, Cretnik A, Dinevski D, Fu FH. Prospective randomized clinical evaluation of conventional single-bundle, anatomic single-bundle, and anatomic double-bundle anterior cruciate ligament reconstruction: 281 cases with 3- to 5-year follow-up. *Am J Sports Med.* 2012;40:512-520.

130. Hwang DH, Shetty GM, Kim JI, et al. Does press-fit technique reduce tunnel volume enlargement after anterior cruciate ligament reconstruction with autologous hamstring tendons? A prospective randomized computed tomography study. *Arthroscopy.* 2013;29:83-88.

131. Ibrahim SA, Abdul Ghafar S, Marwan Y, et al. Intratunnel versus extratunnel autologous hamstring double-bundle graft for anterior cruciate ligament reconstruction: a comparison of 2 femoral fixation procedures. *Am J Sports Med.* 2015;43:161-168.

132. Ibrahim SA, Al-Kussary IM, Al-Misfer AR, Al-Mutairi HQ, Ghafar SA, El Noor TA. Clinical evaluation of arthroscopically assisted anterior cruciate ligament reconstruction: patellar tendon versus gracilis and semitendinosus autograft. *Arthroscopy*. 2005;21:412-417.

133. Ibrahim SA, Hamido F, Al Misfer AK, Mahgoob A, Ghafar SA, Alhran H. Anterior cruciate ligament reconstruction using autologous hamstring double bundle graft compared with single bundle procedures. *J Bone Joint Surg Br.* 2009;91:1310-1315.

134. Indelicato PA, Bittar ES, Prevot TJ, Woods GA, Branch TP, Huegel M. Clinical comparison of freeze-dried and fresh frozen patellar tendon allografts for anterior cruciate ligament reconstruction of the knee. *Am J Sports Med.* 1990;18:335-342.

135. Indelicato PA, Ciccotti MG, Boyd J, Higgins LD, Shaffer BS, Vangsness CT Jr. Aseptically processed and chemically sterilized BTB allografts for anterior cruciate ligament reconstruction: a prospective randomized study. *Knee Surg Sports Traumatol Arthrosc.* 2013;21:2107-2112.

136. Indelicato PA, Linton RC, Huegel M. The results of fresh-frozen patellar tendon allografts for chronic anterior cruciate ligament deficiency of the knee. *Am J Sports Med.* 1992;20:118-121.

137. Ito Y, Deie M, Adachi N, et al. A prospective study of 3-day versus 2-week immobilization period after anterior cruciate ligament reconstruction. *Knee.* 2007;14:34-38.

138. Jacobs CA, Burnham JM, Makhni E, Malempati CS, Swart E, Johnson DL. Allograft augmentation of hamstring autograft for younger patients undergoing anterior cruciate ligament reconstruction. *Am J Sports Med.* 2017;45:892-899.

139. Jadeja H, Yeoh D, Lal M, Mowbray M. Patterns of failure with time of an artificial scaffold class ligament used for reconstruction of the human anterior cruciate ligament. *Knee.* 2007;14:439-442.

140. Jagodzinski M, Geiges B, von Falck C, et al. Biodegradable screw versus a press-fit bone plug fixation for hamstring anterior cruciate ligament reconstruction: a prospective randomized study. *Am J Sports Med.* 2010;38:501-508.

141. Jansson KA, Linko E, Sandelin J, Harilainen A. A prospective randomized study of patellar versus hamstring tendon autografts for anterior cruciate ligament reconstruction. *Am J Sports Med.* 2003;31:12-18.

142. Jenny JY, Clement X. Patient-based decision for resuming activity after ACL reconstruction: a single-centre experience. *Eur J Orthop Surg Traumatol.* 2016;26:929-935.

143. Johnson RJ, Eriksson E, Haggmark T, Pope MH. Five- to ten-year follow-up evaluation after reconstruction of the anterior cruciate ligament. *Clin Orthop Relat Res.* 1984;183:122-140.

144. Jomha NM, Pinczewski LA, Clingeleffer A, Otto DD. Arthroscopic reconstruction of the anterior cruciate ligament with patellar-tendon autograft and interference screw fixation. The results at seven years. *J Bone Joint Surg Br.* 1999;81:775-779.

145. Jonsson H, Elmqvist LG, Kärrholm J, Tegner Y. Over-the-top or tunnel reconstruction of the anterior cruciate ligament? A prospective randomised study of 54 patients. *J Bone Joint Surg Br.* 1994;76:82-87.

146. Järvelä T, Moisala AS, Paakkala T, Paakkala A. Tunnel enlargement after double-bundle anterior cruciate ligament reconstruction: a prospective, randomized study. *Arthroscopy.* 2008;24:1349-1357.

147. Jørgensen U, Bak K, Ekstrand J, Scavenius M. Reconstruction of the anterior cruciate ligament with the iliotibial band autograft in patients with chronic knee instability. *Knee Surg Sports Traumatol Arthrosc.* 2001;9:137-145.

148. Kaeding C, Farr J, Kavanaugh T, Pedroza A. A prospective randomized comparison of bioabsorbable and titanium anterior cruciate ligament interference screws. *Arthroscopy*. 2005;21:147-151.

149. Kamien PM, Hydrick JM, Replogle WH, Go LT, Barrett GR. Age, graft size, and Tegner activity level as predictors of failure in anterior cruciate ligament reconstruction with hamstring autograft. *Am J Sports Med.* 2013;41:1808-1812.

150. Kang HJ, Wang XJ, Wu CJ, Cao JH, Yu DH, Zheng ZM. Single-bundle modified patellar tendon versus double-bundle tibialis anterior allograft ACL reconstruction: a prospective randomized study. *Knee Surg Sports Traumatol Arthrosc.* 2015;23:2244-2249.

151. Karimi-Mobarakeh M, Mardani-Kivi M, Mortazavi A, Saheb-Ekhtiari K, Hashemi-Motlagh K. Role of gracilis harvesting in four-strand hamstring tendon anterior cruciate ligament reconstruction: a double-blinded prospective randomized clinical trial. *Knee Surg Sports Traumatol Arthrosc.* 2015;23:1086-1091.

152. Kautzner J, Kos P, Hanus M, Trc T, Havlas V. A comparison of ACL reconstruction using patellar tendon versus hamstring autograft in female patients: a prospective randomised study. *Int Orthop.* 2015;39:125-130.

153. Khalil LS, Matar RN, Rahman T, et al. Effect of workload after ACL reconstruction on rerupture rates in NBA players. *Orthop J Sports Med.* 2020;8:2325967120964467.

154. Kim SG, Kurosawa H, Sakuraba K, Ikeda H, Takazawa S. The effect of initial graft tension on postoperative clinical outcome in anterior cruciate ligament reconstruction with semitendinosus tendon. *Arch Orthop Trauma Surg.* 2006;126:260-264.

155. Kim SH, Park YB, Kim DH, Pujol N, Lee HJ. Predictive factors for failure of anterior cruciate ligament reconstruction via the trans-tibial technique. *Arch Orthop Trauma Surg.* 2020;140:1445-1457.

156. Kim SJ, Bae JH, Song SH, Lim HC. Bone tunnel widening with autogenous bone plugs versus bioabsorbable interference screws for secondary fixation in ACL reconstruction. *J Bone Joint Surg Am.* 2013;95:103-108.

157. Koga H, Muneta T, Yagishita K, et al. Effect of posterolateral bundle graft fixation angles on clinical outcomes in double-bundle anterior cruciate ligament reconstruction: a randomized controlled trial. *Am J Sports Med.* 2015;43:1157-1164.

158. Koga H, Muneta T, Yagishita K, et al. Mid- to long-term results of single-bundle versus double-bundle anterior cruciate ligament reconstruction: randomized controlled trial. *Arthroscopy.* 2015;31:69-76.

159. Koken M, Akan B, Kaya A, Armangil M. Comparing the anatomic single-bundle versus the anatomic double-bundle for anterior cruciate ligament reconstruction: a prospective, randomized, single blind, clinical study. *Eur Orthop Traumatol.* 2014;5:247-252.

160. Konrads C, Reppenhagen S, Plumhoff P, Hoberg M, Rudert M, Barthel T. No significant difference in clinical outcome and knee stability between patellar tendon and semitendinosus tendon in anterior cruciate ligament reconstruction. *Arch Orthop Trauma Surg.* 2016;136:521-525.

161. Kornblatt I, Warren RF, Wickiewicz TL. Long-term followup of anterior cruciate ligament reconstruction using the quadriceps tendon substitution for chronic anterior cruciate ligament insufficiency. *Am J Sports Med.* 1988;16:444-448.

162. Kösters C, Glasbrenner J, Spickermann L, et al. Repair with dynamic intraligamentary stabilization versus primary reconstruction of acute anterior cruciate ligament tears: 2-year results from a prospective randomized study. *Am J Sports Med.* 2020;48:1108-1116.

163. Laboute E, James-Belin E, Puig PL, Trouve P, Verhaeghe E. Graft failure is more frequent after hamstring than patellar tendon autograft. *Knee Surg Sports Traumatol Arthrosc.* 2018;26:3537-3546.

164. Laoruengthana A, Pattayakorn S, Chotanaputhi T, Kosiyatrakul A. Clinical comparison between six-strand hamstring tendon and patellar tendon autograft in arthroscopic anterior cruciate ligament reconstruction: a prospective, randomized clinical trial. *J Med Assoc Thai.* 2009;92:491-497.

165. Larson CM, Bedi A, Dietrich ME, et al. Generalized hypermobility, knee hyperextension, and outcomes after anterior cruciate ligament reconstruction: prospective, case-control study with mean 6 years follow-up. *Arthroscopy.* 2017;33:1852-1858.

166. Lawhorn KW, Howell SM, Traina SM, Gottlieb JE, Meade TD, Freedberg HI. The effect of graft tissue on anterior cruciate ligament outcomes: a multicenter, prospective, randomized controlled trial comparing autograft hamstrings with fresh-frozen anterior tibialis allograft. *Arthroscopy.* 2012;28:1079-1086.

167. Laxdal G, Kartus J, Eriksson BI, Faxén E, Sernert N, Karlsson J. Biodegradable and metallic interference screws in anterior cruciate ligament reconstruction surgery using hamstring tendon grafts: prospective randomized study of radiographic results and clinical outcome. *Am J Sports Med.* 2006;34:1574-1580.

168. Laxdal G, Kartus J, Hansson L, Heidvall M, Ejerhed L, Karlsson J. A prospective randomized comparison of bone-patellar tendon-bone and hamstring grafts for anterior cruciate ligament reconstruction. *Arthroscopy.* 2005;21:34-42.

169. Leo BM, Krill M, Barksdale L, Alvarez-Pinzon AM. Failure rate and clinical outcomes of anterior cruciate ligament reconstruction using autograft hamstring versus a hybrid graft. *Arthroscopy.* 2016;32:2357-2363.

170. Li J, Wang J, Li Y, Shao D, You X, Shen Y. A prospective randomized study of anterior cruciate ligament reconstruction with autograft, γ-irradiated allograft, and hybrid graft. *Arthroscopy.* 2015;31:1296-1302.

171. Lidén M, Ejerhed L, Sernert N, Laxdal G, Kartus J. Patellar tendon or semitendinosus tendon autografts for anterior cruciate ligament reconstruction: a prospective, randomized study with a 7-Year follow-up. *Am J Sports Med.* 2007;35:740-748.

172. Lind M, Nielsen TG, Soerensen OG, Mygind-Klavsen B, Faunø P. Quadriceps tendon grafts does not cause patients to have inferior subjective outcome after anterior cruciate ligament (ACL) reconstruction than do hamstring grafts: a 2-year prospective randomised controlled trial. *Br J Sports Med.* 2020;54:183-187.

173. Liu Y, Cui G, Yan H, Yang Y, Ao Y. Comparison between single- and double-bundle anterior cruciate ligament reconstruction with 6- to 8-stranded hamstring autograft: a prospective, randomized clinical trial. *Am J Sports Med.* 2016;44:2314-2322.

174. Lord L, Cristiani R, Edman G, Forssblad M, Stålman A. One sixth of primary anterior cruciate ligament reconstructions may undergo reoperation due to complications or new injuries within 2 years. *Knee Surg Sports Traumatol Arthrosc.* 2020;28:2478-2485.

175. Lu W, Wang D, Zhu W, et al. Placement of double tunnels in ACL reconstruction using bony landmarks versus existing footprint remnant: a prospective clinical study with 2-year follow-up. *Am J Sports Med.* 2015;43:1206-1214.

176. Lubowitz JH, Schwartzberg R, Smith P. Cortical suspensory button versus aperture interference screw fixation for knee anterior cruciate ligament soft-tissue allograft: a prospective, randomized controlled trial. *Arthroscopy.* 2015;31:1733-1739.

177. Lubowitz JH, Schwartzberg R, Smith P. Randomized controlled trial comparing all-inside anterior cruciate ligament reconstruction technique with anterior cruciate ligament reconstruction with a full tibial tunnel. *Arthroscopy.* 2013;29:1195-1200.

178. Lund B, Nielsen T, Faunø P, Christiansen SE, Lind M. Is quadriceps tendon a better graft choice than patellar tendon? a prospective randomized study. *Arthroscopy.* 2014;30:593-598.

179. Maletis GB, Cameron SL, Tengan JJ, Burchette RJ. A prospective randomized study of anterior cruciate ligament reconstruction: a comparison of patellar tendon and quadruple-strand semitendinosus/gracilis tendons fixed with bioabsorbable interference screws. *Am J Sports Med.* 2007;35:384-394.

180. Mariscalco MW, Magnussen RA, Mitchell J, et al. How much hamstring graft needs to be in the femoral tunnel? A MOON cohort study. *Eur Orthop Traumatol.* 2015;6:9-13.

181. Marks P, O'Donnell S, Yee G. A pilot clinical evaluation comparing the Mitek bone-tendon-bone cross pin and bioabsorbable screw in anterior cruciate ligament reconstruction fixation, a randomized double blind controlled trial. *Knee*. 2008;15:168-173.

182. Mayr HO, Hochrein A, Hein W, Hube R, Bernstein A. Rehabilitation results following anterior cruciate ligament reconstruction using a hard brace compared to a fluid-filled soft brace. *Knee.* 2010;17:119-126.

183. McCarthy M, Mallett K, Abola M, Vassallo S, Nguyen J. Hospital for Special Surgery ACL Registry: 2-year outcomes suggest low revision and return to OR rates. *HSS J.* 2017;13:119-127.

184. McDevitt ER, Taylor DC, Miller MD, et al. Functional bracing after anterior cruciate ligament reconstruction: a prospective, randomized, multicenter study. *Am J Sports Med.* 2004;32:1887-1892.

185. McGuire DA, Barber FA, Elrod BF, Paulos LE. Bioabsorbable interference screws for graft fixation in anterior cruciate ligament reconstruction. *Arthroscopy.* 1999;15:463-473.

186. McRae S, Leiter J, McCormack R, Old J, MacDonald P. Ipsilateral versus contralateral hamstring grafts in anterior cruciate ligament reconstruction: a prospective randomized trial. *Am J Sports Med.* 2013;41:2492-2499.

187. Mehta VM, Mandala C, Foster D, Petsche TS. Comparison of revision rates in bone-patella tendon-bone autograft and allograft anterior cruciate ligament reconstruction. *Orthopedics.* 2010;33:12.

188. Mei X, Zhang Z, Yang J. Double-layer versus single-layer bone-patellar tendon-bone anterior cruciate ligament reconstruction: a prospective randomized study with 3-year follow-up. *Arch Orthop Trauma Surg.* 2016;136:1733-1739.

189. Meighan AA, Keating JF, Will E. Outcome after reconstruction of the anterior cruciate ligament in athletic patients. A comparison of early versus delayed surgery. *J Bone Joint Surg Br.* 2003;85:521-524.

190. Metso L, Nyrhinen KM, Bister V, Sandelin J, Harilainen A. Comparison of clinical results of anteromedial and transtibial femoral tunnel drilling in ACL reconstruction. *BMC Musculoskelet Disord.* 2020;21:341.

191. Meyers JF, Caspari RB, Cash JD, Manning JB. Arthroscopic evaluation of allograft anterior cruciate ligament reconstruction. *Arthroscopy.* 1992;8:157-161.

192. Meynard P, Pelet H, Angelliaume A, et al. ACL reconstruction with lateral extra-articular tenodesis using a continuous graft: 10-year outcomes of 50 cases. *Orthop Traumatol Surg Res.* 2020;106:929-935.

193. Mirzatolooei F. Comparison of short term clinical outcomes between transtibial and transportal TransFix® femoral fixation in hamstring ACL reconstruction. *Acta Orthop Traumatol Turc.* 2012;46:361-366.

194. Mody BS, Howard L, Harding ML, Parmar HV, Learmonth DJ. The ABC carbon and polyester prosthetic ligament for ACL-deficient knees. Early results in 31 cases. *J Bone Joint Surg Br.* 1993;75:818-821.

195. Mohtadi NG, Chan DS. A randomized clinical trial comparing patellar tendon, hamstring tendon, and double-bundle ACL reconstructions: patient-reported and clinical outcomes at 5-year follow-up. *J Bone Joint Surg Am.* 2019;101:949-960.

196. Moisala AS, Järvelä T, Paakkala A, Paakkala T, Kannus P, Järvinen M. Comparison of the bioabsorbable and metal screw fixation after ACL reconstruction with a hamstring autograft in MRI and clinical outcome: a prospective randomized study. *Knee Surg Sports Traumatol Arthrosc.* 2008;16:1080-1086.

197. Mok DW, Dowd GS. Long-term results of anterior cruciate reconstruction with the patellar tendon. *Injury.* 1993;24:385-388.

198. Spindler KP, Huston LJ, Chagin KM, et al. Ten-year outcomes and risk factors after anterior cruciate ligament reconstruction: A MOON longitudinal prospective cohort study. *Am J Sports Med.* 2018;46:815-825.

199. Muren O, Dahlstedt L, Dalén N. Reconstruction of acute anterior cruciate ligament injuries: a prospective, randomised study of 40 patients with 7-year follow-up. No advantage of synthetic augmentation compared to a traditional patellar tendon graft. *Arch Orthop Trauma Surg.* 2003;123:144-147.

200. Murray MM, Kalish LA, Fleming BC, et al. Bridge-enhanced anterior cruciate ligament repair: two-year results of a first-in-human study. *Orthop J Sports Med.* 2019;7:2325967118824356.

201. Mutsuzaki H, Kanamori A, Ikeda K, Hioki S, Kinugasa T, Sakane M. Effect of calcium phosphate-hybridized tendon graft in anterior cruciate ligament reconstruction: a randomized controlled trial. *Am J Sports Med.* 2012;40:1772-1780.

202. Myers P, Logan M, Stokes A, Boyd K, Watts M. Bioabsorbable versus titanium interference screws with hamstring autograft in anterior cruciate ligament reconstruction: a prospective randomized trial with 2-year follow-up. *Arthroscopy.* 2008;24:817-823.

203. Möller E, Forssblad M, Hansson L, Wange P, Weidenhielm L. Bracing versus nonbracing in rehabilitation after anterior cruciate ligament reconstruction: a randomized prospective study with 2-year follow-up. *Knee Surg Sports Traumatol Arthrosc.* 2001;9:102-108.

204. Nagaraj R, Kumar MN. Revision anterior cruciate ligament reconstruction in the nonathlete population. *Indian J Orthop.* 2019;53:154-159.

205. Nau T, Lavoie P, Duval N. A new generation of artificial ligaments in reconstruction of the anterior cruciate ligament. Two-year follow-up of a randomised trial. *J Bone Joint Surg Br.* 2002;84:356-360.

206. Nazem KHA, Mehrbod M, Borjian A, Sadeghian H. Anterior cruciate ligament recontruction with or without bracing. *Iranian Journal of Medical Sciences.*. 2006;31:151-155.

207. Nelson IR, Chen J, Love R, Davis BR, Maletis GB, Funahashi TT. A comparison of revision and rerupture rates of ACL reconstruction between autografts and allografts in the skeletally immature. *Knee Surg Sports Traumatol Arthrosc.* 2016;24:773-779.

208. Ni QK, Song GY, Zhang ZJ, et al. Steep posterior tibial slope and excessive anterior tibial translation are predictive risk factors of primary anterior cruciate ligament reconstruction failure: a case-control study with prospectively collected data. *Am J Sports Med.* 2020;48:2954-2961.

209. Nicholas SJ, D'Amato MJ, Mullaney MJ, Tyler TF, Kolstad K, McHugh MP. A prospectively randomized double-blind study on the effect of initial graft tension on knee stability after anterior cruciate ligament reconstruction. *Am J Sports Med.* 2004;32:1881-1886.

210. Niu Y, Niu C, Wang X, et al. Improved ACL reconstruction outcome using double-layer BPTB allograft compared to that using four-strand hamstring tendon allograft. *Knee.* 2016;23:1093-1097.

211. Noh JH, Roh YH, Yang BG, Yi SR, Lee SY. Femoral tunnel position on conventional magnetic resonance imaging after anterior cruciate ligament reconstruction in young men: transtibial technique versus anteromedial portal technique. *Arthroscopy.* 2013;29:882-890.

212. Noh JH, Yang BG, Yi SR, Roh YH, Lee JS. Hybrid tibial fixation for anterior cruciate ligament reconstruction with Achilles tendon allograft. *Arthroscopy.* 2012;28:1540-1546.

213. Noojin FK, Barrett GR, Hartzog CW, Nash CR. Clinical comparison of intraarticular anterior cruciate ligament reconstruction using autogenous semitendinosus and gracilis tendons in men versus women. *Am J Sports Med.* 2000;28:783-789.

214. O'Neill DB. Arthroscopically assisted reconstruction of the anterior cruciate ligament. A follow-up report. *J Bone Joint Surg Am.* 2001;83:1329-1332.

215. Offerhaus C, Balke M, Hente J, Gehling M, Blendl S, Höher J. Vancomycin pre-soaking of the graft reduces postoperative infection rate without increasing risk of graft failure and arthrofibrosis in ACL reconstruction. *Knee Surg Sports Traumatol Arthrosc.* 2019;27:3014-3021.

216. Otsuka H, Ishibashi Y, Tsuda E, Sasaki K, Toh S. Comparison of three techniques of anterior cruciate ligament reconstruction with bone-patellar tendon-bone graft. Differences in anterior tibial translation and tunnel enlargement with each technique. *Am J Sports Med.* 2003;31:282-288.

217. Ouanezar H, Blakeney WG, Fernandes LR, et al. Clinical outcomes of single anteromedial bundle biologic augmentation technique for anterior cruciate ligament reconstruction with consideration of tibial remnant size. *Arthroscopy.* 2018;34:714-722.

218. Ovigue J, Bouguennec N, Graveleau N. Arthroscopic anterior cruciate ligament reconstruction is a reliable option to treat knee instability in patients over 50 years old. *Knee Surg Sports Traumatol Arthrosc.* 2020;28:3686-3693.

219. Pandey V, Acharya K, Rao S, Rao S. Femoral tunnel-interference screw divergence in anterior cruciate ligament reconstruction using bone-patellar tendon-bone graft: a comparison of two techniques. *Indian J Orthop*. 2011;45:255-260.

220. Parkes CW, Leland DP, Levy BA, et al. Hamstring autograft anterior cruciate ligament reconstruction using an all-inside technique with and without independent suture tape reinforcement. *Arthroscopy.* 2021;37:609-616.

221. Parkinson B, Robb C, Thomas M, Thompson P, Spalding T. Factors that predict failure in anatomic single-bundle anterior cruciate ligament reconstruction. *Am J Sports Med.* 2017;45:1529-1536.

222. Patel JV, Church JS, Hall AJ. Central third bone-patellar tendon-bone anterior cruciate ligament reconstruction: a 5-year follow-up. *Arthroscopy.* 2000;16:67-70.

223. Patel NM, Talathi NS, Bram JT, DeFrancesco CJ, Ganley TJ. How does obesity impact pediatric anterior cruciate ligament reconstruction? *Arthroscopy.* 2019;35:130-135.

224. Paulos LE, Rosenberg TD, Grewe SR, Tearse DS, Beck CL. The GORE-TEX anterior cruciate ligament prosthesis. A long-term followup. *Am J Sports Med.* 1992;20:246-252.

225. Pennock AT, Ho B, Parvanta K, et al. Does allograft augmentation of small-diameter hamstring autograft ACL grafts reduce the incidence of graft retear? *Am J Sports Med.* 2017;45:334-338.

226. Perelli S, Ibañez F, Gelber PE, Erquicia JI, Pelfort X, Monllau JC. Selective bundle reconstruction in partial ACL tears leads to excellent long-term functional outcomes and a low percentage of failures. *Knee.* 2019;26:1262-1270.

227. Perez JR, Emerson CP, Barrera CM, et al. Patient-reported knee outcome scores with soft tissue quadriceps tendon autograft are similar to bone-patellar tendon-bone autograft at minimum 2-year follow-up: a retrospective single-center cohort study in primary anterior cruciate ligament reconstruction surgery. *Orthop J Sports Med.* 2019;7:2325967119890063.

228. Perkins CA, Busch MT, Christino M, Herzog MM, Willimon SC. Allograft augmentation of hamstring anterior cruciate ligament autografts is associated with increased graft failure in children and adolescents. *Am J Sports Med.* 2019;47:1576-1582.

229. Perrone GS, Webster KE, Imbriaco C, et al. Risk of secondary ACL injury in adolescents prescribed functional bracing after ACL reconstruction. *Orthop J Sports Med.* 2019;7:2325967119879880.

230. Peterson L, Eklund U, Engström B, Forssblad M, Saartok T, Valentin A. Long-term results of a randomized study on anterior cruciate ligament reconstruction with or without a synthetic degradable augmentation device to support the autograft. *Knee Surg Sports Traumatol Arthrosc.* 2014;22:2109-2120.

231. Plaweski S, Cazal J, Rosell P, Merloz P. Anterior cruciate ligament reconstruction using navigation: a comparative study on 60 patients. *Am J Sports Med.* 2006;34:542-52.

232. Price R, Stoney J, Brown G. Prospective randomized comparison of endobutton versus cross-pin femoral fixation in hamstring anterior cruciate ligament reconstruction with 2-year follow-up. *ANZ J Surg.* 2010;80:162-165.

233. Prodromos CC, Han YS, Keller BL, Bolyard RJ. Stability results of hamstring anterior cruciate ligament reconstruction at 2- to 8-year follow-up. *Arthroscopy.* 2005;21:138-146.

234. Pujol N, Colombet P, Potel JF, et al. Anterior cruciate ligament reconstruction in partial tear: selective anteromedial bundle reconstruction conserving the posterolateral remnant versus single-bundle anatomic ACL reconstruction: preliminary 1-year results of a prospective randomized study. *Orthop Traumatol Surg Res.* 2012;98:171-177.

235. Putnis S, Neri T, Grasso S, Linklater J, Fritsch B, Parker D. ACL hamstring grafts fixed using adjustable cortical suspension in both the femur and tibia demonstrate healing and integration on MRI at one year. *Knee Surg Sports Traumatol Arthrosc.* 2020;28:906-914.

236. Rading J, Peterson L. Clinical experience with the Leeds-Keio artificial ligament in anterior cruciate ligament reconstruction. A prospective two-year follow-up study. *Am J Sports Med.* 1995;23:316-319.

237. Rai S, Jin SY, Rai B, et al. A single bundle anterior cruciate ligament reconstruction (ACL-R) using hamstring tendon autograft and tibialis anterior tendon allograft: a comparative study. *Curr Med Sci.* 2018;38:818-826.

238. Rao AJ, Macknet DM, Stuhlman CR, et al. Allograft augmentation of hamstring autograft in anterior cruciate ligament reconstruction results in equivalent outcomes to autograft alone. *Arthroscopy.* 2021;37:173-182.

239. Raviraj A, Anand A, Kodikal G, Chandrashekar M, Pai S. A comparison of early and delayed arthroscopically-assisted reconstruction of the anterior cruciate ligament using hamstring autograft. *J Bone Joint Surg Br.* 2010;92:521-526.

240. Richmond JC, Manseau CJ, Patz R, McConville O. Anterior cruciate reconstruction using a Dacron ligament prosthesis. A long-term study. *Am J Sports Med.* 1992;20:24-28.

241. Risberg MA, Holm I, Steen H, Eriksson J, Ekeland A. The effect of knee bracing after anterior cruciate ligament reconstruction. A prospective, randomized study with two years' follow-up. *Am J Sports Med.* 1999;27:76-83.

242. Risberg MA, Holm I. The long-term effect of 2 postoperative rehabilitation programs after anterior cruciate ligament reconstruction: a randomized controlled clinical trial with 2 years of follow-up. *Am J Sports Med.* 2009;37:1958-1966.

243. Roberts TS, Drez D Jr, McCarthy W, Paine R. Anterior cruciate ligament reconstruction using freeze-dried, ethylene oxide-sterilized, bone-patellar tendon-bone allografts. Two year results in thirty-six patients. *Am J Sports Med.* 1991;19:35-41.

244. Rodríguez-Roiz JM, Sastre-Solsona S, Popescu D, Montañana-Burillo J, Combalia-Aleu A. The relationship between ACL reconstruction and meniscal repair: quality of life, sports return, and meniscal failure rate-2- to 12-year follow-up. *J Orthop Surg Res.* 2020;15:361.

245. Rose MB, Domes C, Farooqi M, Crawford DC. A prospective randomized comparison of two distinct allogenic tissue constructs for anterior cruciate ligament reconstruction. *Knee.* 2016;23:1112-1120.

246. Rosenstiel N, Praz C, Ouanezar H, et al. Combined anterior cruciate and anterolateral ligament reconstruction in the professional athlete: clinical outcomes from the Scientific Anterior Cruciate Ligament Network International Study Group in a series of 70 patients with a minimum follow-up of 2 years. *Arthroscopy.* 2019;35:885-892.

247. Rousseau R, Labruyere C, Kajetanek C, Deschamps O, Makridis KG, Djian P. Complications after anterior cruciate ligament reconstruction and their relation to the type of graft: a prospective study of 958 cases. *Am J Sports Med.* 2019;47:2543-2549.

248. Rowan FE, Huq SS, Haddad FS. Lateral extra-articular tenodesis with ACL reconstruction demonstrates better patient-reported outcomes compared to ACL reconstruction alone at 2 years minimum follow-up. *Arch Orthop Trauma Surg.* 2019;139:1425-1433.

249. Runer A, Csapo R, Hepperger C, Herbort M, Hoser C, Fink C. Anterior cruciate ligament reconstructions with quadriceps tendon autograft result in lower graft rupture rates but similar patient-reported outcomes as compared with hamstring tendon autograft: a comparison of 875 patients. *Am J Sports Med.* 2020;48:2195-2204.

250. Sacramento SN, Magalhães E, Christel P, Ingham S, Fukuda TY. A new technique in double-bundle anterior cruciate ligament reconstruction with implant-free tibial fixation. *Knee Surg Sports Traumatol Arthrosc.* 2016;24:2831-2837.

251. Sajovic M, Vengust V, Komadina R, Tavcar R, Skaza K. A prospective, randomized comparison of semitendinosus and gracilis tendon versus patellar tendon autografts for anterior cruciate ligament reconstruction: five-year follow-up. *Am J Sports Med.* 2006;34:1933-1940.

252. Salem HS, Varzhapetyan V, Patel N, Dodson CC, Tjoumakaris FP, Freedman KB. Anterior Cruciate ligament reconstruction in young female athletes: patellar versus hamstring tendon autografts. *Am J Sports Med.* 2019;47:2086-2092.

253. Salmon L, Russell V, Musgrove T, Pinczewski L, Refshauge K. Incidence and risk factors for graft rupture and contralateral rupture after anterior cruciate ligament reconstruction. *Arthroscopy.* 2005;21:948-957.

254. Salmon LJ, Refshauge KM, Russell VJ, Roe JP, Linklater J, Pinczewski LA. Gender differences in outcome after anterior cruciate ligament reconstruction with hamstring tendon autograft. *Am J Sports Med.* 2006;34:621-629.

255. Saltzman BM, Meyer MA, Weber AE, Poland SG, Yanke AB, Cole BJ. Prospective clinical and radiographic outcomes after concomitant anterior cruciate ligament reconstruction and meniscal allograft transplantation at a mean 5-year follow-up. *Am J Sports Med.* 2017;45:550-562.

256. Sanders TL, Pareek A, Hewett TE, et al. Long-term rate of graft failure after ACL reconstruction: a geographic population cohort analysis. *Knee Surg Sports Traumatol Arthrosc.* 2017;25:222-228.

257. Sarzaeem MM, Najafi F, Razi M, Najafi MA. ACL reconstruction using bone-patella tendon-bone autograft: press-fit technique vs. interference screw fixation. *Arch Orthop Trauma Surg.* 2014;134:955-962.

258. Sasaki S, Tsuda E, Hiraga Y, et al. Prospective randomized study of objective and subjective clinical results between double-bundle and single-bundle anterior cruciate ligament reconstruction. *Am J Sports Med.* 2016;44:855-864.

259. Sastre S, Popescu D, Núñez M, Pomes J, Tomas X, Peidro L. Double-bundle versus single-bundle ACL reconstruction using the horizontal femoral position: a prospective, randomized study. *Knee Surg Sports Traumatol Arthrosc.* 2010;18:32-36.

260. Schlumberger M, Schuster P, Schulz M, et al. Traumatic graft rupture after primary and revision anterior cruciate ligament reconstruction: retrospective analysis of incidence and risk factors in 2915 cases. *Knee Surg Sports Traumatol Arthrosc.* 2017;25:1535-1541.

261. Schroven IT, Geens S, Beckers L, Lagrange W, Fabry G. Experience with the Leeds-Keio artificial ligament for anterior cruciate ligament reconstruction. *Knee Surg Sports Traumatol Arthrosc.* 1994;2:214-218.

262. Scranton PE Jr, Bagenstose JE, Lantz BA, Friedman MJ, Khalfayan EE, Auld MK. Quadruple hamstring anterior cruciate ligament reconstruction: a multicenter study. *Arthroscopy.* 2002;18:715-724.

263. Sevimli R, Gormeli G, Polat H, Kilinc O, Turkmen E, Aslantürk O. Comparison of medium-term revision rates after autograft and allograft anterior cruciate ligament reconstruction. *Ann Ital Chir.* 2020;91:410-416.

264. Sgaglione NA, Del Pizzo W, Fox JM, Friedman MJ, Snyder SJ, Ferkel RD. Arthroscopic-assisted anterior cruciate ligament reconstruction with the semitendinosus tendon: comparison of results with and without braided polypropylene augmentation. *Arthroscopy.* 1992;8:65-77.

265. Shah R, Srinivasan S, Hamed Y, Menon DK. Clinico-radiological outcomes following anatomical anterior cruciate ligament reconstruction using the TransLateral, all-inside technique. *J Clin Orthop Trauma.* 2020;11(Suppl 3):S326-S331.

266. Shaieb MD, Kan DM, Chang SK, Marumoto JM, Richardson AB. A prospective randomized comparison of patellar tendon versus semitendinosus and gracilis tendon autografts for anterior cruciate ligament reconstruction. *Am J Sports Med.* 2002;30:214-220.

267. Shino K, Inoue M, Horibe S, Hamada M, Ono K. Reconstruction of the anterior cruciate ligament using allogeneic tendon. Long-term followup. *Am J Sports Med.* 1990;18:457-465.

268. Shybut TB, Pahk B, Hall G, et al. Functional outcomes of anterior cruciate ligament reconstruction with tibialis anterior allograft. *Bull Hosp Jt Dis.* 2013;71:138-143.

269. Siebold R, Buelow JU, Bös L, Ellermann A. Primary ACL reconstruction with fresh-frozen patellar versus Achilles tendon allografts. *Arch Orthop Trauma Surg.* 2003;123:180-185.

270. Siebold R, Dehler C, Ellert T. Prospective randomized comparison of double-bundle versus single-bundle anterior cruciate ligament reconstruction. *Arthroscopy.* 2008;24:137-145.

271. Singhal MC, Gardiner JR, Johnson DL. Failure of primary anterior cruciate ligament surgery using anterior tibialis allograft. *Arthroscopy.* 2007;23:469-475.

272. Smith PA, Cook CS, Bley JA. All-inside quadrupled semitendinosus autograft shows stability equivalent to patellar tendon autograft anterior cruciate ligament reconstruction: randomized controlled trial in athletes 24 years or younger. *Arthroscopy.* 2020;36:1629-1646.

273. Snow M, Campbell G, Adlington J, Stanish WD. Two to five year results of primary ACL reconstruction using doubled tibialis anterior allograft. *Knee Surg Sports Traumatol Arthrosc.* 2010;18:1374-1378.

274. Sobrado MF, Giglio PN, Bonadio MB, et al. Outcomes after isolated acute anterior cruciate ligament reconstruction are inferior in patients with an associated anterolateral ligament injury. *Am J Sports Med.* 2020;48:3177-3182.

275. Soneru A, Sarwark JF. Survivorship of allograft ACL reconstruction in adolescent patients. *J Orthop.* 2018;16:11-13.

276. Song EK, Seon JK, Yim JH, Woo SH, Seo HY, Lee KB. Progression of osteoarthritis after double- and single-bundle anterior cruciate ligament reconstruction. *Am J Sports Med.* 2013;41:2340-2346.

277. Sonnery-Cottet B, Pioger C, Vieira TD, et al. Combined ACL and anterolateral reconstruction is not associated with a higher risk of adverse outcomes: preliminary results from the SANTI randomized controlled trial. *Orthop J Sports Med.* 2020;8:2325967120918490.

278. Sonnery-Cottet B, Saithna A, Blakeney WG, et al. Anterolateral ligament reconstruction protects the repaired medial meniscus: a comparative study of 383 anterior cruciate ligament reconstructions from the SANTI Study Group with a minimum follow-up of 2 years. *Am J Sports Med.* 2018;46:1819-1826.

279. Sonnery-Cottet B, Saithna A, Cavalier M, et al. Anterolateral ligament reconstruction is associated with significantly reduced ACL graft rupture rates at a minimum follow-up of 2 years: a prospective comparative study of 502 patients from the SANTI Study Group. *Am J Sports Med* . 2017;45:1547-1557.

280. Steadman JR, Matheny LM, Hurst JM, Briggs KK. Patient-centered outcomes and revision rate in patients undergoing ACL reconstruction using bone-patellar tendon-bone autograft compared with bone-patellar tendon-bone allograft: a matched case-control study. *Arthroscopy.* 2015;31:2320-2326.

281. Stener S, Ejerhed L, Sernert N, Laxdal G, Rostgård-Christensen L, Kartus J. A long-term, prospective, randomized study comparing biodegradable and metal interference screws in anterior cruciate ligament reconstruction surgery: radiographic results and clinical outcome. *Am J Sports Med.* 2010;38:1598-1605.

282. Stengel D, Casper D, Bauwens K, Ekkernkamp A, Wich M. Bioresorbable pins and interference screws for fixation of hamstring tendon grafts in anterior cruciate ligament reconstruction surgery: a randomized controlled trial. *Am J Sports Med.* 2009;37:1692-1698.

283. Streich NA, Friedrich K, Gotterbarm T, Schmitt H. Reconstruction of the ACL with a semitendinosus tendon graft: a prospective randomized single blinded comparison of double-bundle versus single-bundle technique in male athletes. *Knee Surg Sports Traumatol Arthrosc.* 2008;16:232-238.

284. Su M, Jia X, Zhang Z, et al. Medium-term (least 5 years) comparative outcomes in anterior cruciate ligament reconstruction using 4SHG, allograft, and LARS ligament. *Clin J Sport Med.* 2021;31:e101-e110.

285. Sun K, Tian S, Zhang J, Xia C, Zhang C, Yu T. Anterior cruciate ligament reconstruction with BPTB autograft, irradiated versus non-irradiated allograft: a prospective randomized clinical study. *Knee Surg Sports Traumatol Arthrosc.* 2009;17:464-474.

286. Sun R, Chen BC, Wang F, Wang XF, Chen JQ. Prospective randomized comparison of knee stability and joint degeneration for double- and single-bundle ACL reconstruction. *Knee Surg Sports Traumatol Arthrosc.* 2015;23:1171-1178.

287. Suomalainen P, Järvelä T, Paakkala A, Kannus P, Järvinen M. Double-bundle versus single-bundle anterior cruciate ligament reconstruction: a prospective randomized study with 5-year results *Am J Sports Med.* 2012;40:1511-1518.

288. Suomalainen P, Moisala AS, Paakkala A, Kannus P, Järvelä T. Double-bundle versus single-bundle anterior cruciate ligament reconstruction: randomized clinical and magnetic resonance imaging study with 2-year follow-up. *Am J Sports Med.* 2011;39:1615-1622.

289. Takazawa Y, Ikeda H, Saita Y, et al. Return to play of rugby players after anterior cruciate ligament reconstruction using hamstring autograft: return to sports and graft failure according to age. *Arthroscopy.* 2017;33:181-189.

290. Tang SP, Wan KH, Lee RH, Wong KK, Wong KK. Influence of hamstring autograft diameter on graft failure rate in Chinese population after anterior cruciate ligament reconstruction. *Asia Pac J Sports Med Arthrosc Rehabil Technol.* 2020;22:45-48.

291. Tay KS, Tan AHC. Clinical outcomes, return to sports, and patient satisfaction after anterior cruciate ligament reconstruction in young and middle-aged patients in an Asian population-a 2-year follow-up study. *Arthroscopy.* 2018;34:1054-1059.

292. Taylor DC, DeBerardino TM, Nelson BJ, et al. Patellar tendon versus hamstring tendon autografts for anterior cruciate ligament reconstruction: a randomized controlled trial using similar femoral and tibial fixation methods. *Am J Sports Med.* 2009;37:1946-1957.

293. Trichine F, Alsaati M, Chouteau J, Moyen B, Bouzitouna M, Maza R. Patellar tendon autograft reconstruction of the anterior cruciate ligament with and without lateral plasty in advanced-stage chronic laxity. A clinical, prospective, randomized, single-blind study using passive dynamic x-rays. *Knee.* 2014;21:58-65.

294. Ugutmen E, Ozkan K, Kilincoglu V, et al. Anterior cruciate ligament reconstruction by using otogenous hamstring tendons with home-based rehabilitation. *J Int Med Res.* 2008;36:253-259.

295. Uribe JW, Arango D, Frank J, Kiebzak GM. Two-year outcome with the AperFix system for ACL reconstruction. *Orthopedics.* 2013;36:e159-e164.

296. Uribe-Echevarria B, Magnuson JA, Amendola A, Bollier MJ, Wolf BR, Hettrich CM. Anterior cruciate ligament reconstruction: a comparative clinical study between adjustable and fixed length suspension devices. *Iowa Orthop J.* 2020;40:121-127.

297. Vadalà AP, Iorio R, De Carli A, et al. An extra-articular procedure improves the clinical outcome in anterior cruciate ligament reconstruction with hamstrings in female athletes. *Int Orthop.* 2013;37:187-192.

298. van Dijck RA, Saris DB, Willems JW, Fievez AW. Additional surgery after anterior cruciate ligament reconstruction: can we improve technical aspects of the initial procedure? *Arthroscopy.* 2008;24:88-95.

299. van Kampen A, Wymenga AB, van der Heide HJ, Bakens HJ. The effect of different graft tensioning in anterior cruciate ligament reconstruction: a prospective randomized study. *Arthroscopy.* 1998;14:845-850.

300. von Essen C, Eriksson K, Barenius B. Acute ACL reconstruction shows superior clinical results and can be performed safely without an increased risk of developing arthrofibrosis. *Knee Surg Sports Traumatol Arthrosc.* 2020;28:2036-2043.

301. Vorlat P, Verdonk R, Arnauw G. Long-term results of tendon allografts for anterior cruciate ligament replacement in revision surgery and in cases of combined complex injuries. *Knee Surg Sports Traumatol Arthrosc.* 1999;7:318-322.

302. Wang HD, Gao SJ, Zhang YZ. Comparison of clinical outcomes after anterior cruciate ligament reconstruction using a hybrid graft versus a hamstring autograft. *Arthroscopy.* 2018;34:1508-1516.

303. Wang F, Kang HJ, Chen BC, Chang YZ, Su LY. Primary ACL reconstruction: comparison of Achilles tendon allograft with tibial anatomical fixation and patellar tendon allograft with external aperture fixation. *Eur J Orthop Surg Traumatol.* 2011;21:333-339.

304. Webb JM, Corry IS, Clingeleffer AJ, Pinczewski LA. Endoscopic reconstruction for isolated anterior cruciate ligament rupture. *J Bone Joint Surg Br.* 1998;80:288-294.

305. Webster KE, Feller JA, Hameister KA. Bone tunnel enlargement following anterior cruciate ligament reconstruction: a randomised comparison of hamstring and patellar tendon grafts with 2-year follow-up. *Knee Surg Sports Traumatol Arthrosc.* 2001;9:86-91.

306. Webster KE, Feller JA, Leigh WB, Richmond AK. Younger patients are at increased risk for graft rupture and contralateral injury after anterior cruciate ligament reconstruction. *Am J Sports Med.* 2014;42:641-647.

307. Webster KE, Feller JA. Exploring the high reinjury rate in younger patients undergoing anterior cruciate ligament reconstruction. *Am J Sports Med.* 2016;44:2827-2832.

308. Wilk RM, Richmond JC. Dacron ligament reconstruction for chronic anterior cruciate ligament insufficiency. *Am J Sports Med.* 1993;21:374-379.

309. Wipfler B, Donner S, Zechmann CM, Springer J, Siebold R, Paessler HH. Anterior cruciate ligament reconstruction using patellar tendon versus hamstring tendon: a prospective comparative study with 9-year follow-up. *Arthroscopy.* 2011;27:653-665.

310. Wolf MR, Murawski CD, van Diek FM, van Eck CF, Huang Y, Fu FH. Intercondylar notch dimensions and graft failure after single- and double-bundle anterior cruciate ligament reconstruction. *Knee Surg Sports Traumatol Arthrosc.* 2015;23:680-686.

311. Woods GA, Indelicato PA, Prevot TJ. The Gore-Tex anterior cruciate ligament prosthesis. Two versus three year results. *Am J Sports Med.* 1991;19:48-55.

312. Wredmark T, Engström B. Five-year results of anterior cruciate ligament reconstruction with the Stryker Dacron high-strength ligament. *Knee Surg Sports Traumatol Arthrosc.* 1993;1:71-75.

313. Xu Y, Ao YF, Wang JQ, Cui GQ. Prospective randomized comparison of anatomic single- and double-bundle anterior cruciate ligament reconstruction. *Knee Surg Sports Traumatol Arthrosc.* 2014;22:308-316.

314. Yabroudi MA, Björnsson H, Lynch AD, et al. Predictors of revision surgery after primary anterior cruciate ligament reconstruction. *Orthop J Sports Med*. 2016;4:2325967116666039.

315. Yagi M, Kuroda R, Nagamune K, Yoshiya S, Kurosaka M. Double-bundle ACL reconstruction can improve rotational stability. *Clin Orthop Relat Res.* 2007;454:100-107.

316. Yasuda K, Kondo E, Ichiyama H, Tanabe Y, Tohyama H. Clinical evaluation of anatomic double-bundle anterior cruciate ligament reconstruction procedure using hamstring tendon grafts: comparisons among 3 different procedures. *Arthroscopy.* 2006;22:240-251.

317. Yoon KH, Kim JS, Kim SJ, Park M, Park SY, Park SE. Eight-year results of transtibial nonanatomic single-bundle versus double-bundle anterior cruciate ligament reconstruction: clinical, radiologic outcomes and survivorship. *J Orthop Surg (Hong Kong).* 2019;27:2309499019840827.

318. Youm YS, Cho SD, Lee SH, Youn CH. Modified transtibial versus anteromedial portal technique in anatomic single-bundle anterior cruciate ligament reconstruction: comparison of femoral tunnel position and clinical results. *Am J Sports Med.* 2014;42:2941-2947.

319. Zaffagnini S, Bruni D, Marcheggiani Muccioli GM, et al. Single-bundle patellar tendon versus non-anatomical double-bundle hamstrings ACL reconstruction: a prospective randomized study at 8-year minimum follow-up. *Knee Surg Sports Traumatol Arthrosc.* 2011;19:390-397.

320. Zaffagnini S, Bruni D, Russo A, et al. ST/G ACL reconstruction: double strand plus extra-articular sling vs double bundle, randomized study at 3-year follow-up. *Scand J Med Sci Sports.* 2008;18:573-581.

321. Zaffagnini S, Grassi A, Marcheggiani Muccioli GM, et al. Return to sport after anterior cruciate ligament reconstruction in professional soccer players. *Knee.* 2014;21:731-735.

322. Zaffagnini S, Grassi A, Romandini I, Marcacci M, Filardo G. Meniscal allograft transplantation combined with anterior cruciate ligament reconstruction provides good mid-term clinical outcome. *Knee Surg Sports Traumatol Arthrosc.* 2019;27:1914-1923.

323. Zaffagnini S, Marcacci M, Lo Presti M, Giordano G, Iacono F, Neri MP. Prospective and randomized evaluation of ACL reconstruction with three techniques: a clinical and radiographic evaluation at 5 years follow-up. *Knee Surg Sports Traumatol Arthrosc.* 2006;14:1060-1069.

324. Zehir S, Zehir R. Suspensory fixation versus novel transverse crosspin for femoral fixation in anterior cruciate ligament reconstruction. *Arch Orthop Trauma Surg.* 2014;134:1579-1585.

325. Zhang J, Ma Y, Pang C, Wang H, Jiang Y, Ao Y. No differences in clinical outcomes and graft healing between anteromedial and central femoral tunnel placement after single bundle ACL reconstruction. *Knee Surg Sports Traumatol Arthrosc.* 2021;29:1734-1741.

326. Zhang Q, Zhang S, Cao X, Liu L, Liu Y, Li R. The effect of remnant preservation on tibial tunnel enlargement in ACL reconstruction with hamstring autograft: a prospective randomized controlled trial. *Knee Surg Sports Traumatol Arthrosc.* 2014;22:166-173.

327. Zhang Z, Gu B, Zhu W, Zhu L, Li Q, Du Y. Arthroscopic single-bundle versus triple-bundle anterior cruciate ligament reconstruction. *Acta Orthop Traumatol Turc.* 2014;48:413-418.

328. Zhang Z, Gu B, Zhu W, Zhu L. Double-bundle versus single-bundle anterior cruciate ligament reconstructions: a prospective, randomized study with 2-year follow-up. *Eur J Orthop Surg Traumatol*. 2014;24:559-565.

329. Zhao J, He Y, Wang J. Double-bundle anterior cruciate ligament reconstruction: four versus eight strands of hamstring tendon graft. *Arthroscopy*. 2007;23:766-770.

330. Ziegler CG, DePhillipo NN, Kennedy MI, Dekker TJ, Dornan GJ, LaPrade RF. Beighton score, tibial slope, tibial subluxation, quadriceps circumference difference, and family history are risk factors for anterior cruciate ligament graft failure: a retrospective comparison of primary and revision anterior cruciate ligament reconstructions. *Arthroscopy*. 2021;37:195-205.
